# The Emerging Role of E3 Ubiquitin Ligase SMURF2 in the Regulation of Transcriptional Co-Repressor KAP1 in Untransformed and Cancer Cells and Tissues

**DOI:** 10.3390/cancers14071607

**Published:** 2022-03-22

**Authors:** Pooja Anil Shah, Sandy Boutros-Suleiman, Andrea Emanuelli, Biagio Paolini, Gal Levy-Cohen, Michael Blank

**Affiliations:** 1 Laboratory of Molecular and Cellular Cancer Biology, Azrieli Faculty of Medicine, Bar-Ilan University, Safed 1311502, Israel; pshah3@mdanderson.org (P.A.S.); boutros@biu.ac.il (S.B.-S.); andrea.emanuelli@u-bordeaux.fr (A.E.); gal.cohen@biu.ac.il (G.L.-C.); 2 Department of Pathology and Laboratory Medicine, IRCCS Fondazione, Istituto Nazionale dei Tumori, 20133 Milan, Italy; biagio.paolini@istitutotumori.mi.it

**Keywords:** SMURF2, KAP1/TRIM28, ubiquitination, interactome, cancer

## Abstract

**Simple Summary:**

KAP1 plays an essential role in different molecular and cellular processes central to carcinogenesis, disease progression, and treatment response, revealing both tumor promoting and anticancer functions. The mechanisms that control the steady-state levels of KAP1 and its protein abundance are not well known. Our findings show that SMURF2, a ubiquitously-expressed HECT-type E3 ubiquitin ligase with suggested anticancer activities, is capable to directly bind, ubiquitinate, and regulate KAP1 expression levels in non-cancerous and tumor cells and tissues. The data further show that SMURF2 has a significant influence on KAP1 interactome, regulating its protein–protein interactions and functions in a catalytically-dependent manner. These findings reveal SMURF2 as a pivotal regulator of KAP1, laying a foundation for the investigation of the role of the SMURF2–KAP1 axis in carcinogenic processes and therapeutic responses to anticancer treatment.

**Abstract:**

KAP1 is an essential nuclear factor acting as a scaffold for protein complexes repressing transcription. KAP1 plays fundamental role in normal and cancer cell biology, affecting cell proliferation, DNA damage response, genome integrity maintenance, migration and invasion, as well as anti-viral and immune response. Despite the foregoing, the mechanisms regulating KAP1 cellular abundance are poorly understood. In this study, we identified the E3 ubiquitin ligase SMURF2 as an important regulator of KAP1. We show that SMURF2 directly interacts with KAP1 and ubiquitinates it in vitro and in the cellular environment in a catalytically-dependent manner. Interestingly, while in the examined untransformed cells, SMURF2 mostly exerted a negative impact on KAP1 expression, a phenomenon that was also monitored in certain *Smurf2*-ablated mouse tissues, in tumor cells SMURF2 stabilized KAP1. This stabilization relied on the unaltered E3 ubiquitin ligase function of SMURF2. Further investigations showed that SMURF2 regulates KAP1 post-translationally, interfering with its proteasomal degradation. The conducted immunohistochemical studies showed that the reciprocal relationship between the expression of SMURF2 and KAP1 also exists in human normal and breast cancer tissues and suggested that this relationship may be disrupted by the carcinogenic process. Finally, through stratifying KAP1 interactome in cells expressing either SMURF2 wild-type or its E3 ligase-dead form, we demonstrate that SMURF2 has a profound impact on KAP1 protein–protein interactions and the associated functions, adding an additional layer in the SMURF2-mediated regulation of KAP1. Cumulatively, these findings uncover SMURF2 as a novel regulator of KAP1, governing its protein expression, interactions, and functions.

## 1. Introduction

Krüppel-associated box (KRAB)-associated protein 1 (KAP1), also known as TRIM28 or TIF1β, is a universal transcriptional co-repressor and chromatin remodeling factor. KAP1 exerts its gene repressive functions by acting as a scaffold protein, interacting with the transcriptional repressors KRAB-ZFPs and chromatin modifiers that epigenetically regulate transcription. These modifiers include the nucleosome remodeling and deacetylase NuRD complex [1], heterochromatin protein HP1 [2], and histone H3 lysine methyltransferase SETDB1 [3]. In addition to transcriptional co-repression, which also includes the silencing of transposable elements such as LINE-1 and Alu [4,5,6,7], KAP1 is engaged in the regulation of several other core biological processes connected to carcinogenesis and therapeutic response. These include cell proliferation, DNA damage response (DDR), autophagy, the degradation of cell energy sensor AMPK and tumor suppressor p53, epithelial-to-mesenchymal transition (EMT), pluripotency maintenance, and immune response, among others [8,9,10,11,12,13,14]. Noteworthy, several of these processes rely on the intrinsic ubiquitin and SUMO E3 ligase activities of KAP1, mediated by its RING and PHD finger domains [14,15,16,17,18].

Clinical investigations mostly suggested KAP1 as a tumor-promoting factor and showed that its overexpression is associated with more aggressive cancer phenotypes and poor patient survival [19]. These associations were documented in breast [20,21], cervical [22], ovarian [23], prostate [24], gastric, and liver cancer [25,26], as well in glioma patients [27,28]. Interestingly, higher expression levels of KAP1 were associated with better overall survival in patients with early stages of lung cancer [29]. Moreover, germline mutations and somatic inactivation of KAP1/TRIM28 were reported in Wilms tumor, the most common type of renal malignancy in childhood [30,31], suggesting the anti-tumorigenic role of KAP1 in these cancers. Despite these proceedings and the accumulating evidence pointing to KAP1 as a potential therapeutic target, the mechanisms regulating KAP1 protein abundance mostly remain elusive.

Here, we report SMURF2, the HECT-type E3 ubiquitin ligase and suggested tumor suppressor, as a novel regulator of KAP1. SMURF2 is a ubiquitously-expressed and evolutionary-conserved E3 ligase whose catalytic activity is implicated in the regulation of diverse molecular and cellular processes. These processes include the regulation of chromatin structure and nuclear shape, DDR and genomic integrity maintenance, gene expression, cell replication, migration, invasion, as well as cancer initiation, progression, and therapeutic response [32,33,34,35,36,37,38,39,40,41,42]. The data that we present in this study show that SMURF2 directly binds, ubiquitinates, and regulates the protein abundance of KAP1 in E3 ligase and cell-context-dependent manners, which is also evident in certain mouse and human normal and cancer tissues. Furthermore, we show that SMURF2 has a significant influence on KAP1 interactome, regulating its protein–protein interactions and functions in a catalytically-dependent manner.

## 2. Materials and Methods

### 2.1. Cell Cultures, Reagents, and Animals

The human embryonic kidney epithelial HEK-293T cells and diploid lung IMR90 fibroblasts were purchased directly from the American Type Culture Collection (ATCC). The human cancer cell lines, including osteosarcoma U2OS cells, cervix carcinoma HeLa cells, prostate carcinoma DU-145 cells, and non-cancerous telomerase-immortalized foreskin BJ1 fibroblasts (BJ1-hTERT) were generously provided by Prof. Yosef Shiloh. Breast carcinoma MDA-MB-468 cells were a gift from Prof. Izhak Haviv. All cell lines, except for the IMR90 cells, were cultured in high glucose DMEM (Biological Industries, Beit-Haemek, Israel) supplemented with 2 mM L-glutamine, 10% (*v*/*v*) fetal bovine serum, and 1% (*v*/*v*) penicillin–streptomycin [34,36,37,38]. IMR90 cells were grown in RPMI 1640 medium (Biological Industries) supplemented with 2 mM L-glutamine, 15% (*v*/*v*) fetal bovine serum, and 1% (*v*/*v*) penicillin–streptomycin.

The proteasomal inhibitor MG-132 and deubiquitinase (DUB) inhibitor N-ethylmaleimide (NEM) were purchased from Merck. 

*Smurf2*-ablated (*Smurf2*KO) and wild-type control C57BL/B6 mice were housed at the SPF animal facility according to the FELASA guidelines and an experimental protocol approved by the Animal Care and Use Committee of Bar-Ilan University (BIU).

### 2.2. Vectors and Constructs

N-terminal FLAG-tagged KAP1 (FLAG-KAP1) was constructed by PCR from GFP-KAP1 construct (a gift from Prof. Shiloh) using the following set of primers: 5′-aaccgaattcgcggcctccgcggcggc-3′ (forward primer containing EcoRI site) and 5′-tatagtcgactcaggggccatcaccagggcca-3′ (reverse primer containing SalI site). The PCR products were then separated and purified from 1% agarose gel, digested with the restriction enzymes EcoRI and SalI (NEB), and ligated into a pRK2-FLAG vector [33]. SMURF2-expressing constructs were generated as previously described [33,36]. All constructs were sequence verified.

### 2.3. Protein Expression and Knockdown

Transient protein expression was carried out by using either polyethyleneimine (PEI, Sigma-Aldrich, St. Louis, MO, USA) or FuGENE^®^6 (E2692, Promega Corporation, Madison, WI, USA) according to the manufacturer’s protocol. GFP-SMURF2 stably expressing cells were generated as described [37]. 

For transient knockdown of SMURF2 expression, predesigned dicer-substrate siRNA duplexes targeting SMURF2, as well as non-silencing (NS) control siRNA, were transfected into cells using either oligofectamine (Invitrogen/Thermo Fisher Scientific, Waltham, MA, USA) or electroporation (Nucleofector^™^, Lonza, Basel, Switzerland), according to manufacturers’ instructions. Knockdown efficiency was assessed 72 h after transfection. The siSMURF2 and siNS sequences are detailed in Table S1. SMURF2 stably knockdown cells were generated by cell infection with lentiviral particles containing pLKO.1-SMURF2-puro vector, following by puromycin selection for at least two weeks [36]. *SMURF2*-depleted cells were also generated using CRISPR/Cas9 genome-editing tool (*SMURF2*^CRISPR^) [43]. 

### 2.4. Western Blot and Immunoprecipitation (IP)

For Western blot analysis, whole cell extracts were obtained by cell lysis in RIPA buffer (50 mM Tris-HCl (pH 7.8), 1% Nonidet P40 substitute, 150 mM NaCl, 0.1% SDS, 0.5% *w*/*v* sodium deoxycholate), supplemented with protease (Roche, Basel, Switzerland) and phosphatase inhibitors (Sigma-Aldrich, St. Louis, MO, USA) cocktails, with subsequent sample sonication (30% amplitude, 1 min on ice) and centrifugation (14,000 rpm, 15 min, 4 °C). The protein extracts from mouse tissues were prepared by tissue homogenization in RIPA buffer using TissueRuptor (Qiagen, Hilden, Germany) and sample sonication. The samples were then cleared by centrifugation. Protein concentration was determined using the Pierce^™^ BCA assay kit (Thermo Fisher Scientific, Waltham, MA, USA). Samples were then resolved in SDS-PAGE and transferred to PVDF membrane (GE Healthcare, Chicago, IL, USA), followed by incubation with the indicated primary antibodies (Table S2) and corresponding horseradish peroxidase-conjugated secondary antibodies (Jackson ImmunoResearch Laboratories, West Grove, PA, USA, 1:10,000). The membranes were then developed using WesternBright ECL HRP substrate (Advansta, San Jose, CA, USA) and visualized in the Syngene G:BOX. Quantification of immunoblots was performed using Gel.Quant.NET, relative to the corresponding loading controls.

For IP and co-IP experiments, cells were lysed in 1% NP40 buffer (10 mM Tris (pH 7.5), 150 mM NaCl, 1% Nonidet P40 substitute, 5 mM EDTA, 10% glycerol). For endogenous co-IP, we used 0.5% NP40 buffer (10 mM Tris (pH 7.5), 150 mM NaCl, 0.5% Nonidet P40 substitute, 5 mM EDTA, 10% glycerol). Both buffers were supplemented with protease and phosphatase inhibitors. Samples were incubated on ice for 30 min, cleared by centrifugation, and protein concentration was assessed using the Pierce^™^ BCA assay kit. For IP of endogenous SMURF2, we used anti-SMURF2 antibody (sc-25511, Santa Cruz, Heidelberg, Germany). Same amount of isotype IgG was used as a control. For IP of recombinant proteins, anti-FLAG M2 affinity gel (F2426, Sigma-Aldrich) and anti-MYC antibody (sc-40, Santa Cruz) were used. Immunoprecipitations were conducted overnight at 4 °C (on rotation). The following day, protein-G Sepharose beads (4 Fast Flow, GE Healthcare, Chicago, IL, USA) were added to the samples, following by sample incubation for additional 2 h on rotation at 4 °C. The beads were then thoroughly washed (at least three times) with an ice-cold lysis buffer and protein complexes eluted from the beads using 5× SDS gel-loading buffer (50 mM Tris-HCl (pH 8), 5 mM EDTA, 5% SDS, 50% glycerol, 50 mM DTT, 0.05% *w*/*v* bromophenol blue, 6% 2-mercaptoethanol) and boiled. The samples were then analyzed in Western blots.

### 2.5. In Vitro Protein Binding and Ubiquitination Assays

These assays were conducted as previously described [36,37,38], with some adjustments. Briefly, for in vitro binding assay, FLAG-KAP1 (produced using the TNT^®^ SP6 high-yield wheat germ expression system (L3260, Promega, Madison, WI, USA) was co-incubated with GST-SMURF2 (purified from bacteria). The reaction was performed in binding buffer (50 mM Tris-HCl (pH 7.5), 120 mM NaCl, 2 mM EDTA, and 0.1% Nonidet P40 substitute) at 37 °C for 15 min. GST-SMURF2 was then pulled down from the reaction using Glutathione Sepharose^™^ 4B beads (GE Healthcare, Chicago, IL, USA), followed by extensive sample washing with an ice-cold binding buffer. The resulting SMURF2–KAP1 complexes were eluted from beads using 5× SDS gel-loading buffer and detected in immunoblots with anti-SMURF2 and anti-KAP1 antibodies. 

For in vitro ubiquitination assay, GST-SMURF2 wild-type (WT), E3 ligase-deficient (C716A; GST-SMURF2*CA*), or GST purified proteins were added to FLAG-KAP1 immobilized on agarose beads (affinity-purified from HEK-293T cells). The mixture was prepared in an E3 ubiquitin ligase reaction buffer (B-71; Boston Biochem Inc., Cambridge, MA, USA) and included HA-ubiquitin (20 µg; U-110, Boston Biochem), ubiquitin-activating enzyme E1 (0.4 µg; E-305, Boston Biochem), ubiquitin-conjugating enzyme E2 (0.6 µg; E2-627-100, Boston Biochem), and ATP-Mg (2 mM; B-20, Boston Biochem). The ubiquitination reaction was performed for 2 h at 37 °C on rotator. Subsequently, the beads were washed four times with an ice-cold buffer (0.5% Nonidet P40 substitute, 25 mM Tris-HCl (pH 7.5), 150 mM NaCl, 2.5 µM ZnSO4, protease inhibitors) and FLAG-KAP1 was eluted by adding to the samples of 5× SDS buffer, followed by sample boiling. The ubiquitination of FLAG-KAP1 was then assessed in immunoblots using anti-HA antibody (71-5500, Invitrogen/Thermo Fisher, Waltham, MA, USA). FLAG-KAP1 and GST-SMURF2 proteins were detected using anti-FLAG^®^ M2 (F3165, Sigma-Aldrich) and anti-SMURF2 (12024, Cell Signaling, Danvers, MA, USA) antibodies.

For the analysis of FLAG-KAP1 ubiquitination in cells (in cellulo ubiquitination assay), HEK-293T cells were transiently transfected with FLAG-KAP1 and the indicated vectors, including HA-ubiquitin and MYC-SMURF2WT or catalytically-inactive SMURF2 (C716G; MYC-SMURF2*CG*). Cells were then lysed in RIPA buffer supplemented with the DUB inhibitor NEM (5 mM) or in 1% SDS, followed by immediate sample boiling at 95 °C for 10–15 min. Samples lysed in 1% SDS were then equilibrated with SDS-free RIPA-NEM buffer to reduce SDS concentration to 0.1%. Subsequently, all samples were sonicated (1 min, 30% amplitude) and FLAG-KAP1 pulled down using anti-FLAG M2 affinity resin. Beads were then extensively washed and the ubiquitination of FLAG-KAP1 analyzed by Western blotting with anti-HA antibody, as described above. For detection of the endogenous KAP1 ubiquitination, KAP1 was IPed from HEK-293T cells using anti-KAP1 antibody (ab109545, Abcam, Cambridge, UK), and then probed with anti-ubiquitin antibody-recognizing endogenous ubiquitin (#58395, Cell Signaling, Boston, MA, USA).

### 2.6. Immunofluorescence (IF) Staining, Proximity Ligation Assay (PLA), and Confocal and Stimulated Emission Depletion (STED) Microscopy

For IF experiments, cells growing on poly-D-lysine-coated glass cover slips were fixed with 4% formaldehyde, permeabilized with 0.5% Triton X-100, and blocked in 3% BSA. Immunostaining was conducted with anti-KAP1 antibody (MA1-2023, Invitrogen/Thermo Fisher, 1:500) and Rhodamine Red^™^-X-conjugated goat anti-mouse secondary antibody (115-296-071, Jackson ImmunoResearch Laboratories, 1:200) for 1 h (for each antibody) at room temperature [36]. DNA was counterstained with Hoechst 33258 (B2883, Sigma-Aldrich) and the coverslips were mounted onto glass slides with ProLong^™^ Diamond antifade mountant (P36961, Invitrogen/Thermo Fisher).

For PLA, U2OS cells growing on coverslips were transiently transfected with either MYC-SMURF2 or MYC empty vector. Next day, the PLA staining was performed using the Duolink^™^ in situ Red Starter Kit Mouse/Rabbit (DUO92101, Sigma-Aldrich) with anti-MYC (#2278S, Cell Signaling, 1:200) and anti-KAP1 (MA1-2023, Invitrogen/Thermo Fisher, 1:500) antibodies. The fluorescent images were then visualized and captured using a LSM780 inverted confocal microscope (Zeiss, Jena, Germany) fitted with a Plan-Apochromat 63X/1.40 Oil DIC M27 objective. The images were analyzed using ZEN Blue (version 2.3 lite) and ImageJ (NIH) software tools. All comparative images in IF and PLA assays were obtained under identical microscope and camera settings.

For STED microscopy, U2OS cells expressing GFP-SMURF2 were immunostained using anti-KAP1 antibody (MA1-2023, Invitrogen/Thermo Fisher, 1:500) and Rhodamine Red^™^-X-conjugated goat anti-mouse IgG (115-296-071, Jackson ImmunoResearch Laboratories, 1:500). STED images were captured using Leica SPi 8 Super-Resolution gSTED inverted confocal microscope fitted with HC PL APO 100×/1.40 Oil objective. The obtained images were analyzed using the Leica Application Suite X software [37]. ImageJ (NIH) was used to quantify areas of overlap/colocalization between GFP-SMURF2 and KAP1.

### 2.7. Immunohistochemistry (IHC) and Tissue Microarray (TMA) Analysis

These analyses were performed as we previously described [33,38,44], with some modifications. In brief, the tissues obtained from *Smurf2*KO (*Smurf2*^−/−^) and littermate WT control mice were fixed in 4% paraformaldehyde, embedded in paraffin blocks, and sectioned using Leica RM2235 microtome to prepare 5 μm tissue sections. Human normal and breast cancer TMAs (FDA999m and BR804a, respectively) were purchased from US Biomax, Inc (Rockville, MD, USA). Immunohistochemical staining was conducted using anti-KAP1 (A300-274A, Bethyl laboratories, Montgomery, TX, USA, 1:1000) and anti-SMURF2 (sc-25511, Santa Cruz, 1:100) antibodies. All comparable samples were layered on the same slide and all staining procedures were carried out on horizontally positioned samples. Images were captured using Axio Scan.Z1 (Zeiss) through Plan-Apochromat 20x/0.8 M27 objective. The comparative images were acquired under identical settings. TMAs were scored for staining intensity and percentage of positively-stained cells by an experienced pathologist—Biagio Paolini (Istituto Nazionale dei Tumori, Milan, Italy)—using the standard scoring system: 0 ≤ 10%; 1 = 10–24%; 2 = 25–49%; 3 = 50–74%; 4 = 75–100%.

### 2.8. KAP1 Interactome Analysis

The immunoprecipitated KAP1 protein complexes (purified through the FLAG-KAP1 pull-down) were eluted using 8 M urea buffer (8 M urea, 20 mM Tris (pH 7.5), 10 mM DTT, 100 mM NaCl) and subjected for mass spectrometry (MS) analysis at the Smoler Protein Research Center (Technion, Israel), where the samples were trypsin-digested and analyzed using LC-MS/MS in Q Exactive^™^ Plus mass spectrometer (Thermo Fisher). The resulting peptides were identified by Discoverer^™^ software against human proteome using two search algorithms: Sequest (Thermo Fisher) and Mascot (Matrix Sciences, Sherwood, OR, USA). All identified peptides were filtered with high confidence (false discovery rate (FDR) ≤ 1%), top rank, mass accuracy, and a minimum coverage of two peptides. An additional filter of two-fold change of the area under the peak of the peptides (a measure of a protein abundance) was used to enrich KAP1 interactors in the analyzed samples. The resulting KAP1 interactors were subjected to gene ontology (GO) analysis that was carried out using ToppFun suite bioinformatics tool [37,45]. GO terms with Benjamini and Hochberg-adjusted FDR (q-value FDR B&H) < 0.05 were considered as significant. Protein classification was performed using the PANTHER platform, and the protein–protein interaction network reconstructed using the STRING tool [37,46,47]. Only interactors that are connected within the network were considered and k-means clustering method was applied to classify proteins into different categories. The proteomic data were also analyzed using QIAGEN Ingenuity pathway analysis (IPA) (QIAGEN Inc., https://digitalinsights.qiagen.com/IPA, accessed on 7 February 2022) [48]. This analysis was conducted on a total number of KAP1-associated proteins identified at FDR ≤1%, with Fisher’s exact *p*-value (−log(*p*-value)) set on ≥1.3. A negative fold change expression value was considered as downregulated, whereas a positive value was considered upregulated. Positive and negative z-scores were used to predict pathway activation or inhibition, respectively.

### 2.9. Quantitative Real Time PCR (qRT-PCR)

Total RNA was extracted from cells using RNeasy mini kit (Qiagen) according to the manufacturer’s instructions. The cDNA was synthesized from total RNA with random primers using the High Capacity cDNA Reverse Transcription Kit (4368814, Applied Biosystems, Waltham, MA, USA). Equal amounts of synthesized cDNA (100 ng/reaction) were subjected to PCR analysis in ViiA^™^ 7 Real-Time PCR System (Thermo Fisher) using Fast SYBR™ Green Master Mix (4385612, Applied Biosystems). KAP1 cDNA levels were calculated using 2^−ΔΔCT^ and normalized to GAPDH gene expression. The sequence of primers used for qRT-PCR analysis is shown in Table S3. The primers were calibrated for their specificity to the exon–exon spanning region through the standard curve method.

### 2.10. Statistical Analyses

Two-tailed Student’s *t*-test was used for statistical analysis of the data obtained in IF, PLA, IHC, qRT-PCR, and ubiquitination assays. Data with *p*-values less than 0.05 were considered as statistically significant.

## 3. Results

### 3.1. KAP1 Is a Novel SMURF2 Interactor

**Figure 1:**
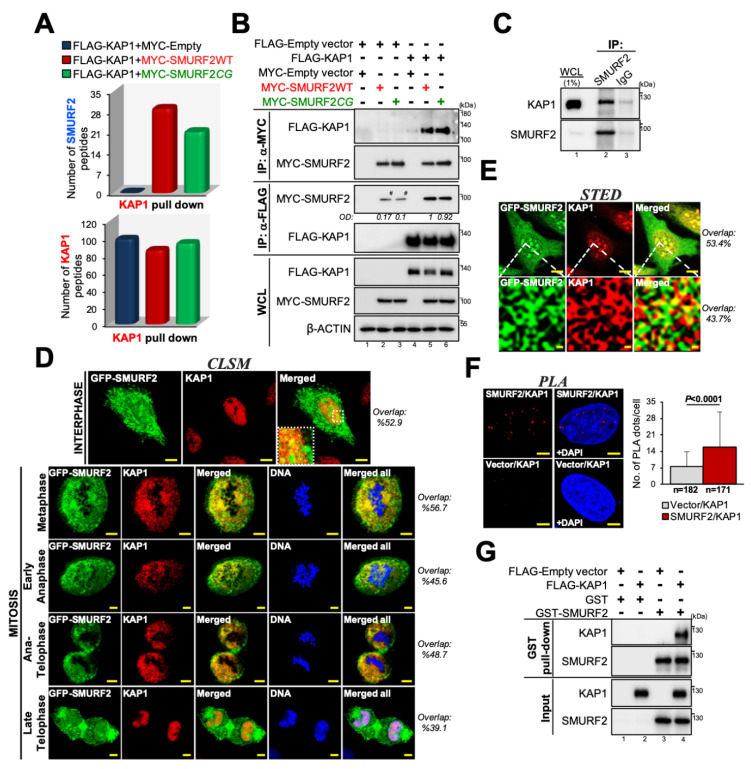
SMURF2 and KAP1 directly interact with each other. (**A**) The number of SMURF2 and KAP1 peptides identified in FLAG-KAP1 immunoprecipitates in HEK-293T cells using MS. SMURF2WT—wild type SMURF2; SMURF2*CG*—catalytically-inactive SMURF2 (C716G). (**B**) Reciprocal co-IP analysis showing KAP1 in complex with SMURF2 and vice versa in HEK-293T cells. WCL, whole cell lysates. ^#^-non-specific. (**C**) Endogenous co-IP showing interaction between endogenous SMURF2 and KAP1 proteins in HEK293T cells. Isotype IgG was used as a control. (**D**) Confocal laser scanning microscopy (CLSM) images showing co-localization of GFP-SMURF2 and endogenous KAP1 in interphase and mitotic U2OS cells. DNA was counterstained with Hoechst 33258. Scale bars: 10 µm and 5 µm for interphase and mitotic cells, respectively. (**E**) STED microscopy images showing co-localization of KAP1 and GFP-SMURF2 in the U2OS cell nucleus. White rectangles mark the area detailed under the STED microscopy. Scale bars: 10 µm and 40 nm in the upper and bottom images, respectively. (**F**) PLA showing the sites of direct protein–protein interaction of endogenous KAP1 and MYC-SMURF2 in the U2OS cell nucleus (red signal). Cells transfected with MYC-Empty vector served as a control. Scale bars: 5 µm. Quantification of the SMURF2-KAP1 PLA data is shown on the right. In total, 171–182 cells were quantified from at least 10 different fields. Data are mean ± SD. (**G**) In vitro GST-pull down assay showing a direct interaction between purified GST-SMURF2 and FLAG-KAP1. The uncropped Western blot images can be found in Figure S6.

In our preliminary studies aimed to characterize the interactome of SMURF2, we noted that KAP1 may be associated with SMURF2 [44]. This association was also suggested by the involvement of these proteins in similar molecular and cellular processes, including gene expression, DDR, and chromatin remodeling. To investigate the possibility that KAP1 is a bona fide interactor of SMURF2 and, potentially, its substrate, we conducted several lines of investigation. First, we co-expressed FLAG-KAP1 and MYC-SMURF2 (wild-type, SMURF2WT, or its catalytically-inactive form, SMURF2*CG*) in HEK-293T cells, immunoprecipitated FLAG-KAP1 with anti-FLAG resin and analyzed the samples using MS. The analysis showed that KAP1 is associated with both SMURF2WT and SMURF2*CG* (Figure 1A). Particularly, we found 29 and 21 SMURF2-specific peptides (in SMURF2WT and SMURF2*CG* expressing samples, respectively) in a complex with KAP1 versus zero peptides in FLAG-KAP1 and MYC-Empty co-expressing cells (Figure 1A, upper panel). The total number of KAP1 peptides identified in these samples was comparable: 99 in the MYC-Empty sample, 86 in SMURF2WT, and 94 in the SMURF2*CG* samples (Figure 1A, bottom panel). Reciprocal co-IP analysis validated these findings and showed that KAP1 interacts with both the SMURF2WT and *CG* form, and vice versa (Figure 1B). The binding detected between endogenous proteins provided further confirming evidence that KAP1 and SMURF2 are interacting partners (Figure 1C). This interaction was not limited to HEK-293T cells but was also observed in other types of cells. Particularly, using U2OS cells, we demonstrate that SMURF2 and KAP1 co-immunoprecipitate and co-localize in both interphase and mitotic cells (Figure 1D and Figure S1A–C). The conducted super-resolution STED microscopy analysis showed that these proteins interact within the nanometer scale range (Figure 1E), suggesting a physical interaction between SMURF2 and KAP1. This finding was further corroborated by the proximity ligation assay that detected a direct interaction between these proteins in the cellular milieu (Figure 1F). The direct interaction between SMURF2 and KAP1 was also evident in the in vitro binding assay, wherein we used purified GST-SMURF2 and FLAG-KAP1 proteins (Figure 1G).

### 3.2. KAP1 Is a Direct Ubiquitination Substrate of SMURF2

**Figure 2:**
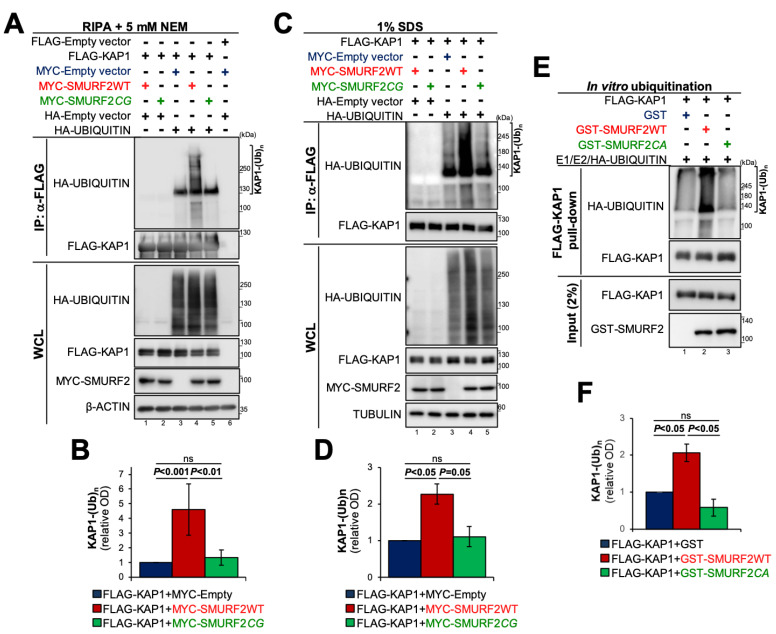
KAP1 is a direct ubiquitination substrate of SMURF2. (**A**) In cellulo ubiquitination assay showing SMURF2-mediated ubiquitination of KAP1 and its dependency on E3 ligase activity of SMURF2. HEK-293T cells. Cells were lysed in RIPA buffer supplemented with the deubiquitinase inhibitor NEM. (**B**) Quantification of the data shown in (**A**) obtained in six different experiments. Data are mean ± SD. ns—non-significant. (**C**) In cellulo ubiquitination assay following cell lysis in 1% SDS. HEK-293T cells. (**D**) Quantification of the data presented in (**C**) obtained from two independent experiments. Data are mean ± SD. (**E**) In vitro ubiquitination assay showing ubiquitination of purified FLAG-KAP1 by GST-SMURF2. Catalytically-inactive GST-SMURF2*CA* and GST were used as additional controls. (**F**) Quantification of the results obtained in two separate in vitro ubiquitination assays. Data are mean ± SD. The uncropped Western blot images can be found in Figure S6.

Subsequently, we proceeded to examine the possibility that SMURF2 operates as an E3 ubiquitin ligase of KAP1. To this end, we first conducted in cellulo ubiquitination assays, involving co-expression of FLAG-KAP1, MYC-SMURF2 (either WT or its inactive form), and HA-ubiquitin in HEK-293T cells. Cells transfected with the corresponding empty vectors served as additional controls. Following transfection, cells were lysed in RIPA buffer supplemented with the DUB inhibitor NEM and sonicated to ensure the complete extraction of KAP1 from the RIPA-resistant fractions. FLAG-KAP1 was then pulled-down, and its ubiquitination analyzed in immunoblots using anti-HA antibody, which specifically recognizes HA-ubiquitin conjugated to FLAG-KAP1 (Figure 2A, lanes 3–5 vs. 1, 2, and 6). The results presented in the figure also showed that in cells co-expressing FLAG-KAP1, HA-ubiquitin, and MYC-Empty vector, KAP1 predominantly exhibits a monoubiquitination pattern (Figure 2A, lane 3). The addition to the cells of a catalytically-active SMURF2 significantly increased the oligo/polyubiquitination of KAP1 (Figure 2A, lane 4), while the expression of SMURF2*CG* showed results highly similar to the control, MYC-Empty, sample (Figure 2A, lane 5), suggesting that SMURF2 ubiquitinates KAP1 in an E3 ligase-dependent manner. These results were consistently observed in multiple independent experiments (Figure 2B).

Similar findings were also obtained in cells lysed with 1% SDS, followed by immediate sample boiling and sonication (Figure 2C,D). This stringent cell lysis condition permits more efficient deactivation of cellular DUBs, removing their possible interference with the ubiquitination of KAP1. It also enables the disruption of protein–protein interactions formed in the cellular milieu, eliminating the possibility that the observed ubiquitination of KAP1 emanates from its co-IPed partners. Nevertheless, independently of cell lysis conditions, we consistently observed SMURF2-mediated ubiquitination of KAP1 and its dependence on SMURF2 E3 ligase activity. Additionally, we detected the endogenous ubiquitination of KAP1 facilitated by the adventitious expression of SMURF2 (Figure S2A).

Finally, to demonstrate that KAP1 is a direct ubiquitination target of SMURF2, we conducted the ubiquitination reconstitution assay (in vitro ubiquitination assay), using purified components of the ubiquitination cascade. These components included ubiquitin-activating enzyme E1, ubiquitin conjugating enzyme E2, GST-SMURF2 (either WT or its E3 ligase-dead form), HA-ubiquitin, and purified FLAG-KAP1 as a substrate. Following the incubation, FLAG-KAP1 was pulled-down from the reaction and its ubiquitination analyzed using anti-HA antibody. The results (Figure 2E,F) show that SMURF2 is capable to directly ubiquitinate KAP1 in an E3 ligase-dependent manner, whereas SMURF2 mutant failed to produce this phenomenon, revealing results highly similar to the GST control. The purity of the proteins, which we produced and used in this study, was verified by Coomassie gel staining (Figure S2B).

### 3.3. SMURF2 Positively Regulates the Protein Abundance of KAP1 in Cancer Cells

**Figure 3:**
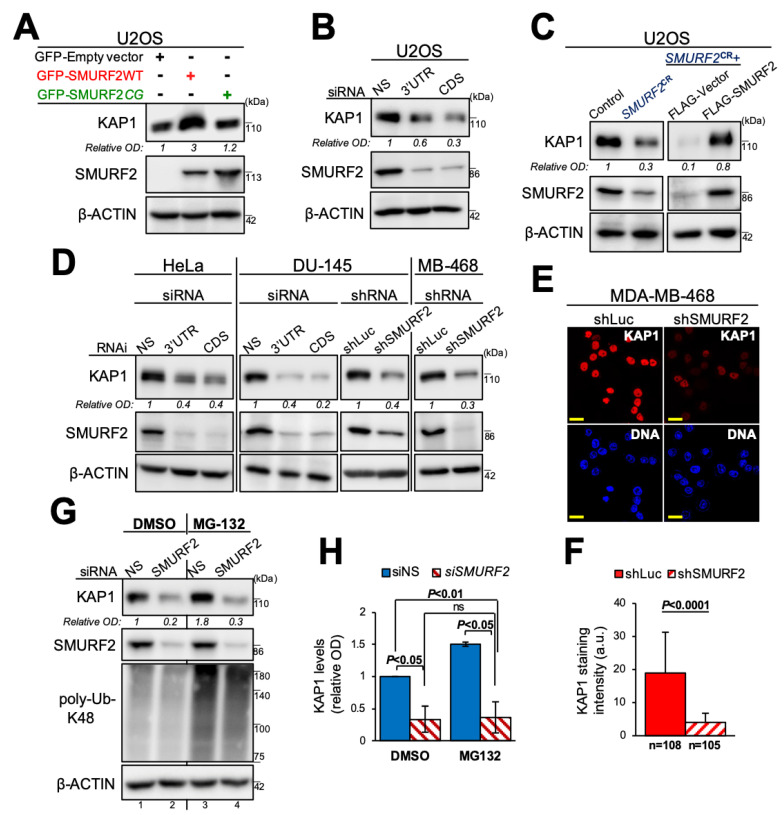
SMURF2 positively regulates KAP1 protein levels in tumor cells. (**A**) Western blot analysis showing that overexpression of SMURF2WT, but not its E3 ligase-inactive mutant (SMURF2*CG*), enhances the protein abundance of KAP1 in osteosarcoma U2OS cells. (**B**) Immunoblot analysis showing diminished protein levels of KAP1 in SMURF2 knockdown U2OS cells. Two different siRNAs targeting *SMURF2* mRNA expression at either 3′UTR or coding sequence (CDS) were used. NS—non-silencing siRNA. (**C**) Immunoblot analysis of KAP1 expression in SMURF2^CRISPR^ knockdown U2OS cells (*SMURF2*^CR^, left panels) and in cells following SMURF2 reconstitution (right panels). SMURF2^CR^ cells were reconstituted with FLAG-SMURF2. Cells transduced with an empty FLAG vector were used as a control. (**D**) Western blot analysis showing the effect of SMURF2 knockdown on KAP1 protein levels in different types of human cancer cells: cervix carcinoma HeLa cells, prostate carcinoma DU-145 cells, and breast carcinoma MDA-MB-468 cells. Non-silencing siNS and shLuc were used as controls for siRNA and shRNA experiments, respectively. (**E**,**F**) Confocal microscopy analysis of KAP1 expression in SMURF2 knockdown MDA-MB-468 cells. Scale bars: 20 µm. Quantification of the results (panel F) is shown as mean ± SD. n—number of cells quantified for each group from 10 different fields. (**G**) Western blot analysis of KAP1 protein expression in SMURF2 knockdown U2OS cells treated with the proteasomal inhibitor MG-132 (5 µM; 4 h). The inhibition of the proteasomal pathway by MG-132 was verified using anti-poly-ubiquitin-K48-specific antibody. (**H**) Quantification of data shown in (**G**) from two independent experiments. Data are mean ± SD. The uncropped Western blot images can be found in Figure S6.

To determine the consequence of SMURF2 binding to KAP1 and its ubiquitination, we first analyzed the effect of the enforced expression of SMURF2 on the steady-state levels of KAP1. The results show that KAP1 protein levels were markedly increased in SMURF2WT-expressing cells, as compared to the cells transduced with either an empty vector or SMURF2 mutant (Figure 3A), suggesting that SMURF2 upregulates KAP1 expression in a catalytically-dependent manner. Conversely, the knocking down of SMURF2 through RNAi dramatically decreased the expression levels of KAP1 (Figure 3B). Similar results were also obtained following SMURF2 depletion using the CRISPR/Cas9 gene-editing tool (*SMURF2^CR^*), disrupting *SMURF2* expression at the genetic level (Figure 3C, left panels). Furthermore, we found that subsequent reconstitution of active SMURF2 into *SMURF2^CR^* cells reversed this phenomenon and elevated the expression level of KAP1 diminished by *SMURF2* depletion (Figure 3C, right panels). This finding suggests that loss of SMURF2 was solely responsible for the decreased cellular abundance of KAP1 in *SMURF2^CR^* cells. The reduced protein levels of KAP1 following SMURF2 depletion were also observed in other cancer cell models, including cervix carcinoma HeLa cells, prostate carcinoma DU-145 cells, and breast adenocarcinoma MDA-MB-468 cells. This effect was monitored both after acute and stable SMURF2 knockdown using different approaches (Figure 3D–F).

Further investigations revealed that mRNA levels of KAP1 were not significantly changed in SMURF2-depleted cells (Figure S3), suggesting that SMURF2 regulates KAP1 post-transcriptionally/post-translationally. The post-translational level of KAP1 regulation by SMURF2 was further corroborated using the proteasomal inhibitor MG-132, which increased the protein levels of KAP1 in SMURF2-proficient cells (Figure 3G, lane 3 vs. 1) but failed to do so in SMURF2 knockdown samples (Figure 3G, lane 4 vs. 2, and Figure 3H).

### 3.4. SMURF2 Negatively Regulates KAP1 Expression in Untransformed Human Cells and Mouse Tissues

**Figure 4:**
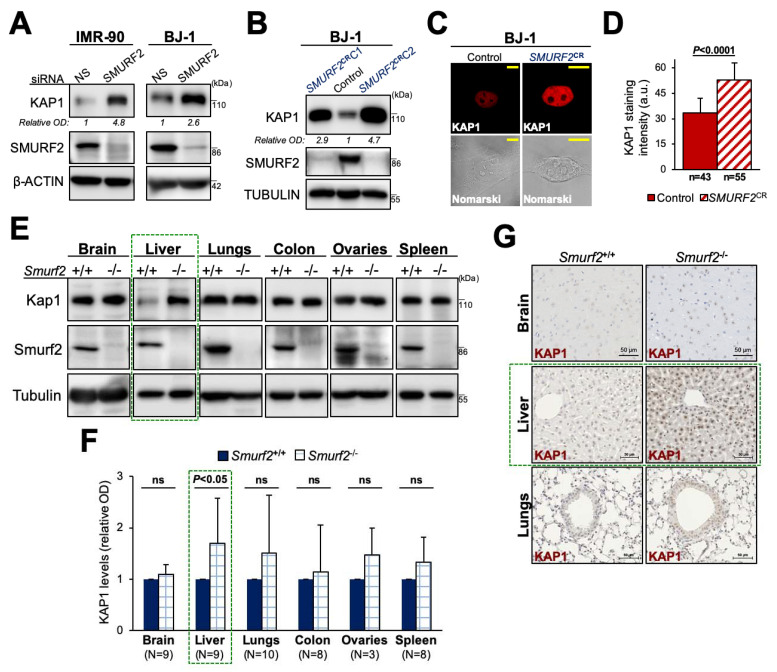
SMURF2 exerts a negative impact on KAP1 expression in untransformed human cells and normal mouse tissues. (**A**) Western blot analyses of KAP1 expression in IMR-90 and BJ-1 cells knocked down of SMURF2. NS—non-silencing siRNA. (**B**) Immunoblot analysis of KAP1 expression in SMURF2 knock-out (*SMURF2*^CR^) BJ1 cells. Two different SMURF2^CRISPR^ clones (*SMURF2*^CR^C1 and *SMURF2*^CR^C2) were examined. (**C**,**D**) Confocal microscopy analysis of KAP1 protein expression in *SMURF2*^CR^ BJ1 cells. Scale bars: 10 µm. Quantification of the results (panel D) is presented as mean ±S D. n—number of cells quantified for each group from at least 10 different fields. (**E**) Western blot analysis of KAP1 expression in *Smurf2*KO (*Smurf2*^−/−^) and littermate control wild-type (*Smurf2*^+/+^) mouse tissues. (**F**) Quantification of data shown in (**E**) obtained in different pairs of mice. Data are mean ± SD. N—number of animals. (**G**) IHC staining of KAP1 (brown) in *Smurf2*WT and KO mouse tissues. The nuclei were counterstained with hematoxylin (blue). Scale bars: 50 µm. The uncropped Western blot images can be found in Figure S6.

As SMURF2 was reported to play a differential role in malignant and normal cells [32,44], we examined the effect of SMURF2 depletion on KAP1 expression in human untransformed cell lines. To this end, we knocked down SMURF2 in diploid lung IMR-90 fibroblasts and dermal BJ1 cells, which are known to maintain the untransformed phenotype typical of normal cells. The expression levels of KAP1 in these cells were then analyzed by Western blotting. The results (Figure 4A) showed that in contrast to tumor cells SMURF2 knockdown in IMR-90 and BJ1 cells markedly increased KAP1 protein levels, suggesting that, in these cells, SMURF2 operates as a negative regulator of KAP1 expression. This effect was also observed in *SMURF2*KO (*SMURF2^CR^*) cells (Figure 4B–D). Noteworthy, the data showed that SMURF2 exhibits its regulatory effect on KAP1 in a cell-context-dependent manner, as we did not detect any significant changes in KAP1 expression levels in SMURF2 knockdown MCF-10A mammary epithelial cells (data not shown). The context-dependent regulation of KAP1 by SMURF2 was also noted in *Smurf2*-ablated mouse tissues. Specifically, we found that Kap1 levels were significantly elevated in *Smurf2*KO liver tissues but showed no significant increase in other analyzed organs and tissues (Figure 4E–G).

### 3.5. The SMURF2–KAP1 Relationship in Human Normal and Cancer Tissues

**Figure 5:**
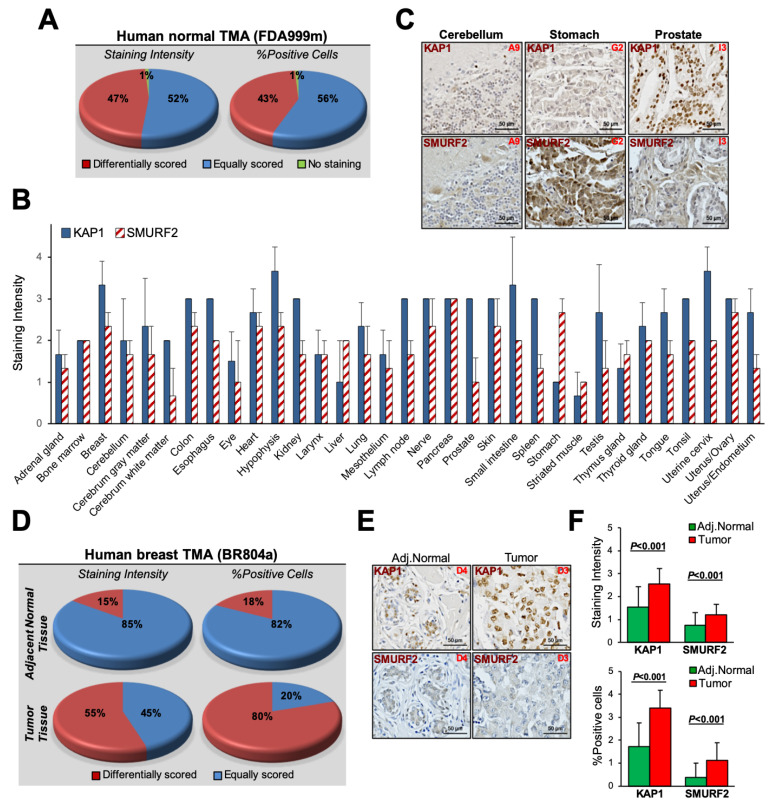
Analysis of the SMURF2–KAP1 relationship in human normal and cancer tissues. (**A**) Summary of the quantification analysis of SMURF2 and KAP1 staining intensity and percentage of positive cells in a panel of human normal tissues (FDA999m). Note, some of the tissues in this TMA are cancer-adjacent normal tissues. The full details on this TMA are available at https://www.biomax.us/FDA999m, accessed on 22 February 2022. (**B**) SMURF2 and KAP1 expression in different tissue types. Data are mean ± SD. (**C**) Representative IHC images of SMURF2 and KAP1-stained normal tissues. A9 exemplifies tissues in which KAP1 and SMURF2 were equally scored (i.e., cerebellum), and G2 and I3 show differentially scored samples: low KAP1 (intensity score ≤ 1.5) and high SMURF2 (intensity score ≥ 2) in stomach, and high KAP1 and low SMURF2 in prostate tissue samples. The nuclei were counterstained with hematoxylin (blue). Scale bars: 50 µm. (**D**) Summary of the quantification analysis of SMURF2 and KAP1 staining intensity and %positive cells in human normal and breast cancer TMA (BR804a). (**E**) Representative images of IHC staining of SMURF2 and KAP1 in breast TMA. D3 and D4 are the coordinates of the samples in the tissue array. Scale bars: 50 µm. Adj.Normal—adjacent normal tissue. (**F**) The relationship between scoring values of SMURF2 and KAP1 in breast TMA. Data are mean ± SD obtained from the analysis of 40 tumor and corresponding normal tissues [44].

To relate our findings to the clinical settings, we examined the relationship between the protein expression of KAP1 and SMURF2 in different human normal and breast cancer tissues, using tissue microarrays (TMAs) and IHC analysis. Particularly, we stained and analyzed two different sets of TMAs: a multi-organ normal TMA (FDA999m, US Biomax), containing 32 types of normal tissues taken from three individuals, and breast cancer TMA (BR804a, US Biomax), containing 40 cases of breast cancer samples with 40 matched adjacent normal tissues. The IHC analysis of normal tissues revealed that, despite the heterogenous expression of SMURF2 and KAP1 in different individuals, ~50% of tissues were scored equally in terms of the SMURF2 and KAP1 staining intensity and the percentage of positive cells, meaning that tissues with a lower expression of SMURF2 showed a lower expression of KAP1, and vice versa (Figure 5A). These tissues included the cerebellum, adrenal gland, pancreas, thyroid, and bone marrow, among others (Figure 5B,C). In the lymph node, spleen, stomach, and prostate, SMURF2 and KAP1 were differentially scored: samples with a lower expression of SMURF2 showed a higher expression of KAP1, and samples with higher SMURF2 levels exhibited low KAP1 expression.

The IHC analysis of breast TMA showed that, in more than 80% of normal breast tissues, SMURF2 and KAP1 were equally scored in terms of staining intensity and the percentage of positive cells (Figure 5D, upper panels). In cancer tissues, however, the SMURF2–KAP1 relationship was considerably shifted towards the differential score (Figure 5D, bottom panels). Most of the cancer tissues showed either a lower expression of SMURF2 and higher expression of KAP1 (Figure 5E) or the opposite: a higher expression of SMURF2 and lower expression of KAP1, suggesting that the carcinogenic process may change the relationship between the expression of these proteins. Noteworthy, and similar to the previous findings [20,21,44], the expression levels SMURF2 and KAP1 in tumors were considerably higher as compared to their normal matching counterparts (Figure 5F). These IHC findings, together with our data showing that in certain types of cancer cells, including breast cancer, KAP1 is stabilized by SMURF2, may imply that elevated expression of KAP1 in certain tumors emanates from the heightened expression of SMURF2. 

### 3.6. SMURF2 Has a Significant Influence on KAP1 Interactome, Regulating Its Protein Interactions and Downstream Functions in an E3 Ligase-Dependent Manner

**Figure 6:**
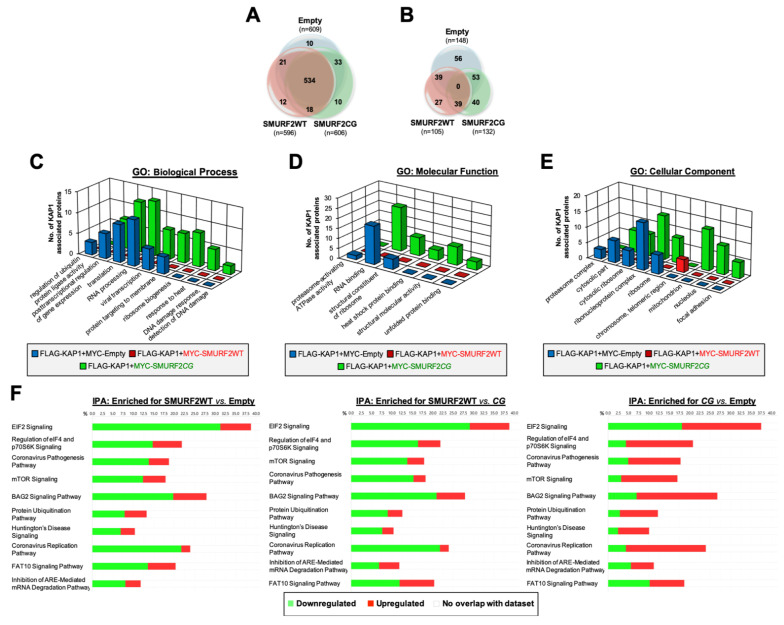
SMURF2 influences KAP1 interactome, regulating its functions in an E3 ligase-dependent manner. (**A**) Venn diagram showing a total number of KAP1-associated proteins (n) identified in HEK-293T cells using MS. All identified peptides were filtered with FDR ≤ 1%, top rank, mass accuracy and a minimum coverage of two peptides. (**B**) Number of KAP1-associated proteins which abundance changed ≥2-fold. (**C**–**E**) GO analysis of biological processes (panel C), molecular functions (panel D), and cellular components (panel E) of KAP1-interacting partners affected by SMURF2. GO terms were considered significant when showing Benjamini and Hochberg adjusted FDR (q-value FDR B&H) < 0.05. The detailed results are shown in Table S4. (**F**) KAP1 interactome analysis using IPA. Top ten signaling pathways with the highest −log(*p*-value) are shown. The detailed list/analysis of all signaling pathways predicted to be altered by SMURF2WT vs. SMURF2*CG* and control empty vector samples are shown in Table S5.

To further investigate the impact of SMURF2 on KAP1, we then stratified the cellular interactome of KAP1 in cells expressing either active SMURF2 or its catalytically-deficient mutant. The analysis showed that KAP1 interacts with ~600 proteins, with 534 proteins shared between the analyzed groups: SMURF2WT, SMURF2CG, and the control MYC-Empty (Figure 6A). Applying an additional threshold of ≥2-fold change in the protein abundance reduced the number of KAP1 interactors to 105–148 proteins, with the lowest number of KAP1-interacting partners detected in SMURF2WT cells (Figure 6B). Additionally, the results revealed that the enforced expression of SMURF2WT abolished KAP1 interactions with 56 proteins. Along with this finding, the MS data showed that the expression of both SMURF2WT and SMURF2CG forms increased KAP1 associations with its several known binding partners and promoted the formation of previously unreported protein–protein interactions of KAP1 (PXD029642). In total, 27 and 40 unique KAP1-interacting proteins were identified in cells expressing SMURF2WT and SMURF2*CG*, respectively (Figure 6B).

The protein class analysis using the PANTHER classification system showed that the KAP1 binding partners in a control sample consisted nucleic acid metabolism proteins, gene-specific transcriptional regulators, translational proteins, protein-modifying enzymes, chromatin-binding or regulatory proteins, and scaffolds/adaptors, among several others (Figure S4A). Similar classes of proteins were also detected in SMURF2-enriched cells, although with certain differences. For example, in cells with the enforced expression of SMURF2WT, we did not find as KAP1 interactors the scaffold/adaptor proteins, which were present in both control and SMURF2*CG*-expressing samples (Figure S4B,C). Instead, SMURF2WT-expressing cells showed the enrichment of protein-binding activity modulators, suggesting that this group of proteins was enriched in KAP1 interactome by the expression of active SMURF2. 

The reconstruction of the KAP1 protein–protein interaction map using the STRING tool showed that SMURF2 upregulates the molecular networks associated with the negative regulation of transcription, mRNA splicing, RNA binding, processing, protein translation and targeting, as well as heat shock protein binding (Figure S5A). In parallel, its decreased KAP1 associations with molecular networks implicated in the regulation of gene expression, RNA binding, splicing, mRNA stability, as well as structural molecule activity and regulation of cell cycle phase transition (Figure S5B). Moreover, the gene ontology (GO) analysis revealed that, in comparison to an empty control and catalytically-inactive SMURF2*CG*, SMURF2WT downregulated several key biological processes and molecular functions associated with the activities of KAP1 interactome. These included RNA binding and processing, viral transcription, post-transcriptional regulation of gene expression, and translation, among others (Figure 6C,D and Table S4). Furthermore, the GO:cellular component analysis indicated that SMURF2WT markedly decreased the association of KAP1 interactors with the ribonucleoprotein complexes, cytosolic components, and ribosomes, while increasing the KAP1 association with proteins associated with the chromosome telomeric region (Figure 6E). Additionally, the ingenuity pathway analysis (IPA) of the KAP1 interactome in SMURF2WT vs. SMURF2*CG* and the empty control samples revealed significant differences in the percentage of enrichment of the proteins involved in key signaling cellular pathways. These included EIF2 signaling, regulation of eIF4 and p70S6K signaling, mTOR signaling, the BAG2 signaling pathway, and others (Figure 6F and Table S5). Most of these pathways were predicted to be downregulated by SMURF2WT, recapitulating findings of the GO analysis on the downregulation of biological processes and the molecular functions of KAP1 interactors by SMURF2. Taken together, these findings show that SMURF2 has a significant influence on KAP1 interactome, regulating its functions and associated signaling pathways in a catalytically-dependent manner.

## 4. Discussion

Accumulating evidence shows that KAP1 is intrinsically engaged in several core biological processes pertinent to carcinogenesis, disease progression, therapeutic response, and patient survival, revealing both tumor-promoting and suppressing activities. Despite these proceedings, the KAP1 regulatory mechanisms, especially the mechanism regulating its cellular abundance, remain poorly characterized. Up-to-date, there are only two studies related to the regulation of protein abundance of KAP1 in cells. The first study showed that following DNA damage the phosphorylated and SUMOylated KAP1 is targeted for ubiquitin-mediated proteasomal degradation facilitated by RING-type E3 ubiquitin ligase RNF4 [49]. The second, more recent research, reported that Dcaf11, one of the central components in the cullin-4-based RING E3 ligase complex, targets KAP1 for ubiquitin-mediated degradation, thereby regulating telomere elongation in early embryos and embryonic stem cells [50].

In our study, we expanded the list of KAP1 post-translational regulators and identified the HECT-type E3 ubiquitin ligase SMURF2 as a pivotal regulator of KAP1. We showed that SMURF2 and KAP1 directly interact with each other, both in vitro and in the cellular environment (Figure 1), allowing a direct ubiquitin transfer from SMURF2 to KAP1 (Figure 2). We further demonstrated that this phenomenon is relied on unaltered E3 ubiquitin ligase activity of SMURF2, leading to the stabilization of KAP1 in cancer cells (Figure 3A). Depletion of *SMURF2* using either the RNAi or CRISPR/Cas9-based approach profoundly diminished the protein expression of KAP1 (Figure 3B–D), while unaffecting its mRNA expression levels (Figure S3). Noteworthy, the re-expression of SMURF2 in *SMURF2*-depleted cells restored KAP1 protein levels (Figure 3C), suggesting SMURF2 as one of the major factors controlling the stability of KAP1 through the proteasomal degradation pathway (Figure 3G,H). Interestingly, while acting in cancer cells as a KAP1 stabilizing factor, in untransformed IMR90 and BJ1 cells, SMURF2 showed a negative impact on KAP1 expression (Figure 4A–D), the phenomenon that was also observed in certain *Smurf2*KO tissues (Figure 4E–G). This suggested that SMURF2 regulates KAP1 in cell/tissue-specific manner. The contextual nature of KAP1 regulation by SMURF2 was also evident from the examination of several other untransformed and cancer cell strains, including mammary epithelial MCF-10A cells, breast carcinoma MDA-MB-231 cells, and prostate adenocarcinoma PC3 cells. In all these cells, SMURF2 knockdown did not affect the steady-state levels of KAP1 (data not shown) in contrast to IMR-90 and BJ1 cells, as well as to the MDA-MB-468, HeLa, DU-145, and U2OS cells shown in this study (Figure 3 and Figure 4). The context-dependent regulation of KAP1 by SMURF2 was also suggested by the IHC results obtained in TMAs (Figure 5). One of the possible explanations for the differential regulation of KAP1 expression by SMURF2 in different types of cells and tissues could be the activation of compensatory mechanisms, including alterations in the cell transcriptional activity, affecting KAP1 levels. Indeed, SMURF2 was previously reported to act as a pleiotropic factor affecting gene expression at both epigenetic and post-translational levels in a cell-context-dependent manner [32,34,51]. Another potential explanation for the contextual regulation of KAP1 by SMURF2 could emanate from the differential expression in these cells of the deubiquitinating enzymes (DUBs). Indeed, the expression and activities of DUBs were extensively documented to impose a significant impact on protein degradation dynamics and proteome remodeling, both under normal conditions and in the disease states, including cancer [52,53]. In the case of SMURF2, at least two different DUBs–USP4 and USP15, shown to interfere with SMURF2-mediated ubiquitination of its protein targets [54,55], might be implicated. Additional possibilities may involve the ubiquitination of distinct lysine residues of KAP1 by SMURF2 and/or the formation of different ubiquitin chains on KAP1 in different types of cells and tissues. Further investigations of these possibilities will be needed for understanding the full details of the relationship between KAP1 and SMURF2. The subsequent studies should also determine the biological consequences of the SMURF2-mediated regulation of KAP1 interactome and its associated functions and signaling pathways uncovered in this work (Figure 6). Particularly, the influence of SMURF2–KAP1 on RNA biology, post-transcriptional regulation of gene expression, protein translation, structural molecule activity, regulation of the cell cycle, and telomere maintenance. Addressing these questions will allow better understanding of the role of the SMURF2–KAP1 complex in the biology of untransformed and malignant cells, promoting the development of pathway-oriented precision therapies targeting KAP1 in cancer.

## 5. Conclusions

In this work, we identified the HECT-type E3 ubiquitin ligase SMURF2 as an important cellular factor regulating the protein expression of KAP1 in different types of cells and tissues in a context-dependent manner. These findings lay a foundation for further investigations of the role of the SMURF2–KAP1 axis in the carcinogenic process and therapeutic response, with an ultimate goal to develop new, more effective treatment strategies based on the disruption in cancers of the dysregulated SMURF2/KAP1 module.

## Data Availability

The mass spectrometry proteomics data of KAP1 interactome have been deposited to the ProteomeXchange Consortium via the PRIDE [56] partner repository with the dataset identifier PXD029642, available online: https://www.ebi.ac.uk/pride/archive, accessed on 10 November 2021.

## Supplementary Materials

The following supporting information can be downloaded at: https://www.mdpi.com/article/10.3390/cancers14071607/s1, Figure S1: SMURF2 and KAP1 physically interact with each other, Figure S2: Endogenous ubiquitination assay and Coomassie gel staining of purified proteins used in this study, Figure S3: qRT-PCR analysis of KAP1 mRNA expression in SMURF2 knock down cells, Figure S4: PANTHER analysis of protein classes of KAP1 interactors, Figure S5: STRING analysis of protein-protein interaction network of KAP1 interactors affected by SMURF2, Figure S6: Uncropped Western Blot (WB) images, Table S1: siRNA sequences, Table S2: Primary antibodies used for immunoblotting, Table S3: qRT-PCR primers used in the study, Table S4: Details of GO terms for KAP1-interacting proteins changed in abundance by SMURF2, Table S5: Details of the Ingenuity Pathway Analysis (IPA) of KAP1 interactome affected by SMURF2WT and its catalytically-inactive mutant (SMURF2*CG*).

## Abbreviations

AMPK	AMP-activated protein kinase
CDS	coding sequence
co-IP	co-immunoprecipitation
Dcaf11	Ddb1- and Cul4-associated factor 11
DDR	DNA damage response
DsiRNA	dicer-substrate siRNA
DTT	dithiothreitol
DUB	deubiquitinase
EMT	epithelial-to-mesenchymal transition
FDR	false discovery rate
GAPDH	glyceraldehyde-3-phosphate dehydrogenase
GO	gene ontology
GST	glutathione-S-transferase
HA	hemagglutinin
HECT	homologous to the E6-AP carboxyl terminus
HP1	heterochromatin protein 1
IF	immunofluorescence
IHC	immunohistochemistry
IP	immunoprecipitation
IPA	ingenuity pathway analysis
IPed	immunoprecipitated
KAP1	Krüppel-associated box (KRAB)-associated protein 1
KO	knock-out
KRAB-ZFPs	Krüppel-associated box (KRAB) domain-containing zinc-finger proteins
LINE-1	long interspersed nuclear element 1
MS	mass spectrometry
NEM	*N*-ethylmaleimide
NS	non-silencing
NuRD	nucleosome remodeling deacetylase
PEI	polyethylenimine
PEPs	posterior error probabilities
PHD	plant homeodomain
PLA	proximity ligation assay
RING	really interesting new gene
SD	standard deviation
SMURF2	Smad ubiquitin regulatory factor 2
STED	stimulated emission depletion microscopy
SUMO	small ubiquitin-like modifier
TIF1β	transcriptional intermediary factor 1β
TRIM28	tripartite motif-containing protein 28
USP	ubiquitin-specific peptidase
WCL	whole cell lysate
