# Supplementary material for: The Emerging Role of E3 Ubiquitin Ligase SMURF2 in the Regulation of Transcriptional Co-Repressor KAP1 in Untransformed and Cancer Cells and Tissues
Source: Cancers (Basel). 2022 Mar 22;14(7):1607. doi: 10.3390/cancers14071607 (PMC8997158; doi:10.3390/cancers14071607)
Supplement: Supplementary file 1 [file cancers-14-01607-s001.zip › cancers-1637887-supplementary.pdf]

Supplementary Materials: The Emerging Role of E3 Ubiquitin Ligase SMURF2 in the Regulation of Transcriptional Co-repressor KAP1 in Untransformed and Cancer Cells and Tissues

Pooja Anil Shah, Sandy Boutros-Suleiman, Andrea Emanuelli, Biagio Paolini, Gal Levy-Cohen and Michael Blank

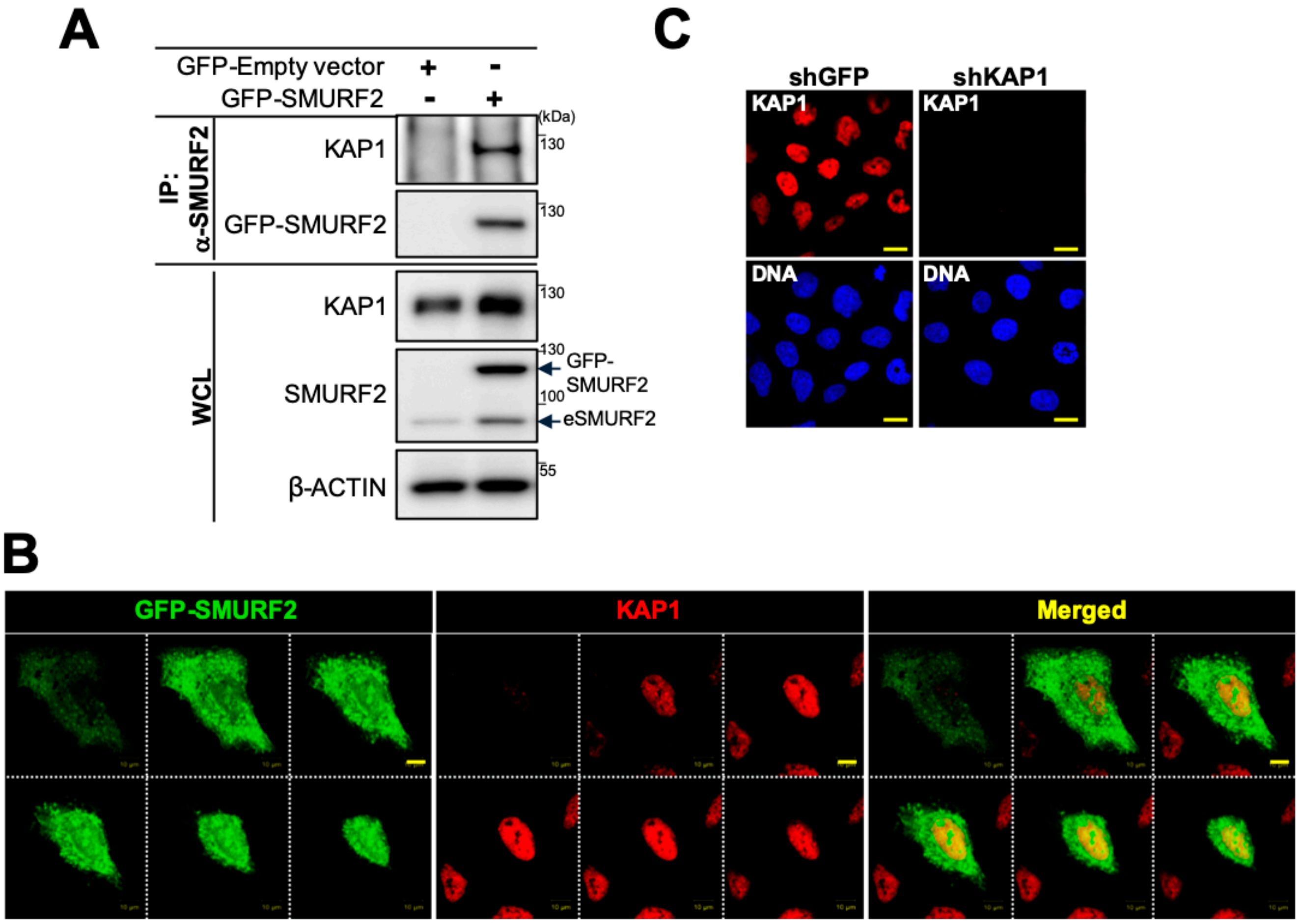

Figure S1. SMURF2 and KAP1 physically interact with each other. (**A**) co-IP analysis showing interaction between GFP-SMURF2 and endogenous KAP1 in U2OS cells. GFP-Empty vector was used as a control. eSMURF2—endogenous SMURF2. WCL, whole cell lysates. (**B**) Z-Stack analysis of co-localization of GFP-SMURF2 and endogenous KAP1 in the U2OS cell nucleus. Scale bars: 10 µm. (**C**) Validation of the specificity of anti-KAP1 antibody using shKAP1 U2OS cells. Cells transduced with shGFP vector served as a control. Scale bars: 20 µm. The uncropped Western blot images can be found in Figure S6.

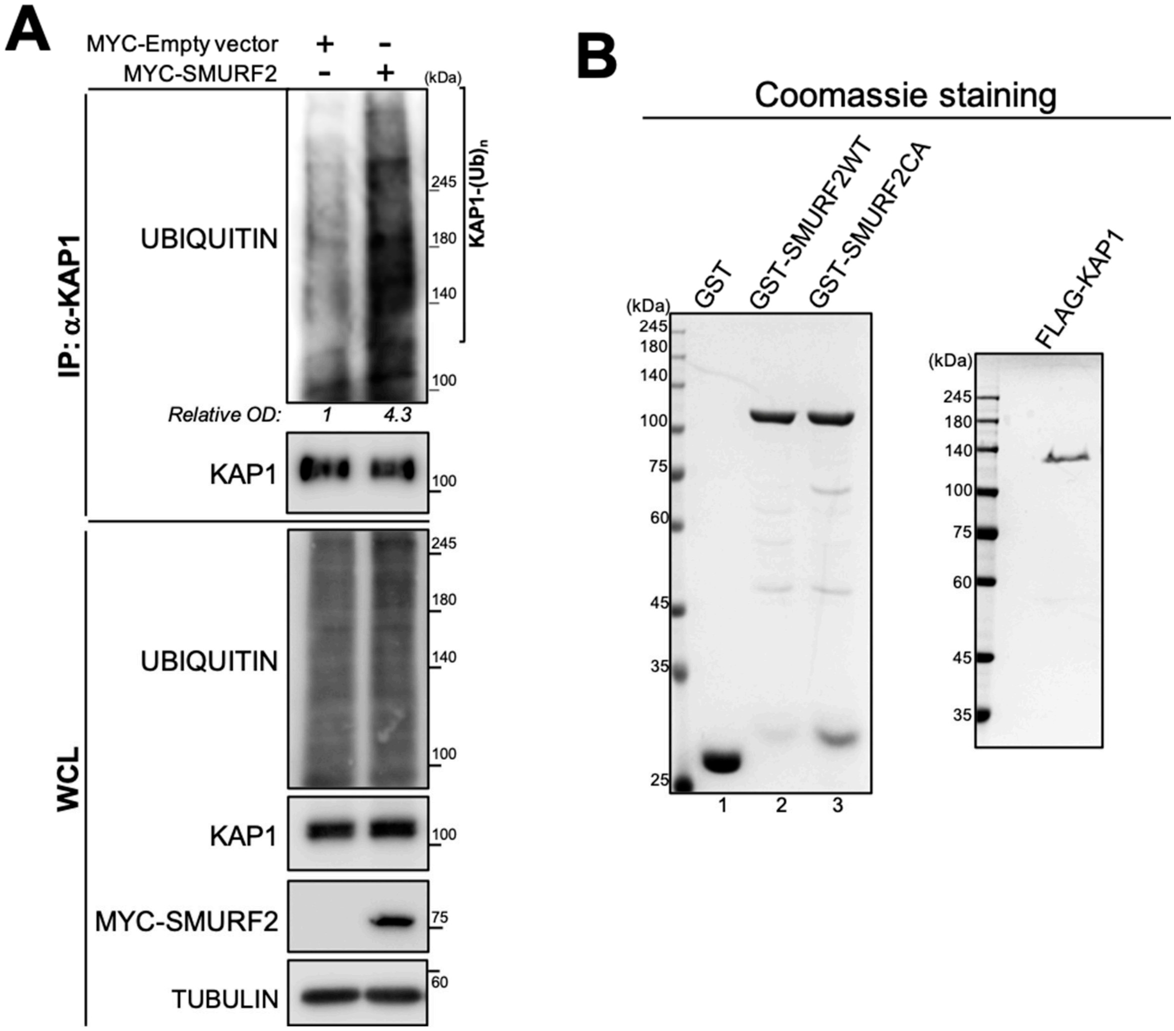

Figure S2. Endogenous ubiquitination assay and Coomassie gel staining of purified proteins used in this study. (**A**) In cellulo ubiquitination assay showing SMURF2-mediated ubiquitination of endogenous KAP1. KAP1 was IPed from HEK-293T cells transiently transfected with either MYC-SMURF2 or MYC empty-vector, and probed with anti-ubiquitin antibody (#58395, Cell Signaling) recognizing endogenous ubiquitin. (**B**) Coomassie gel staining of purified GST, GST-SMURF2 and FLAG-KAP1. The uncropped Western blot images can be found in Figure S6.

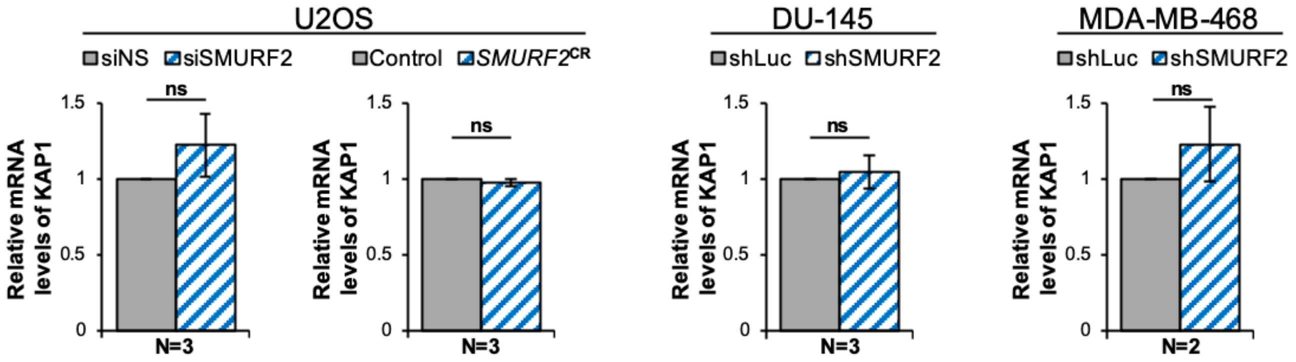

Figure S3. qRT-PCR analysis of *KAP1* mRNA expression in SMURF2 knock down cells. Data are mean ±SD. N–number of biological replicates with three technical replicates in each experiment. ns–non-significant.

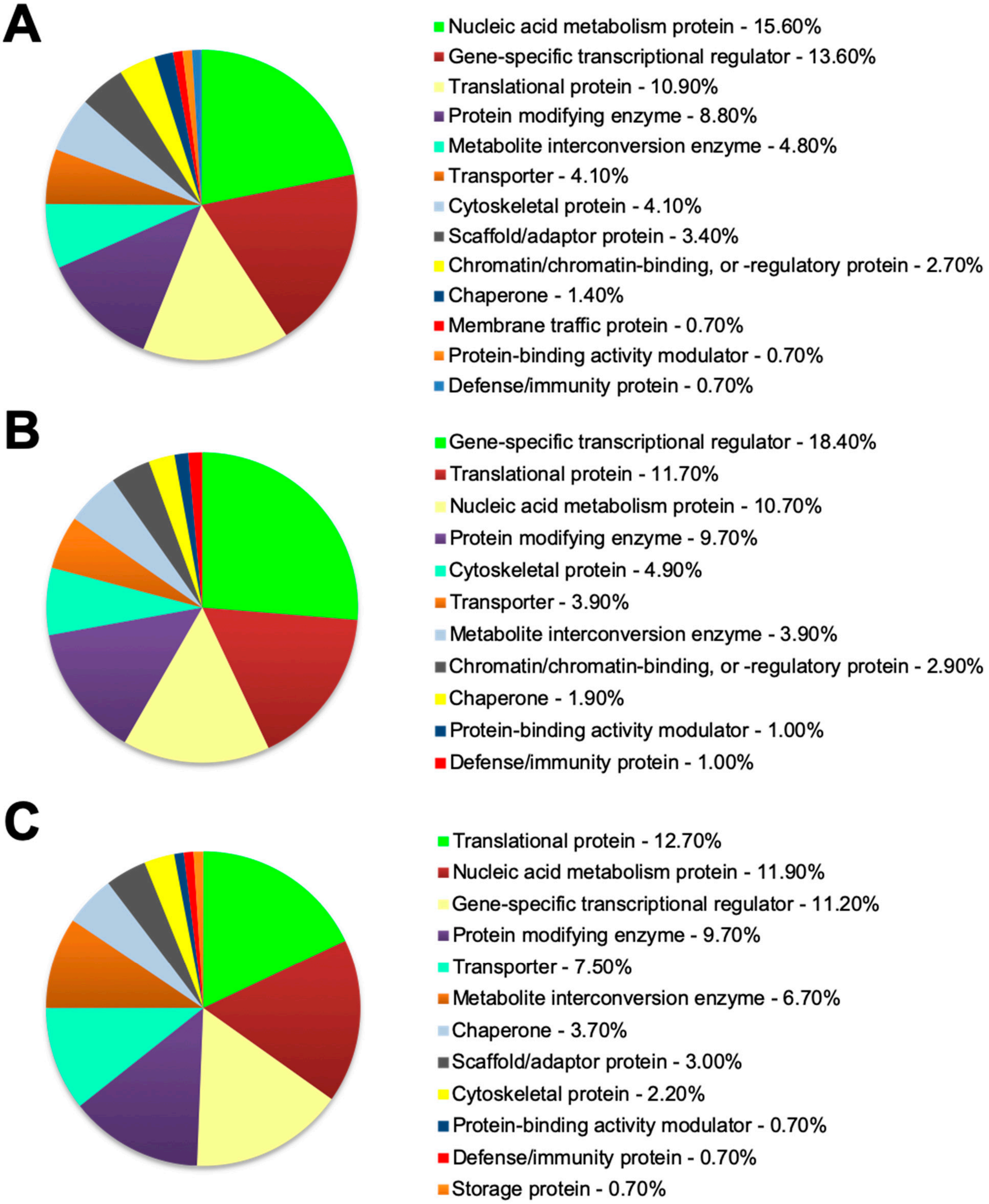

Figure S4. PANTHER analysis of protein classes of KAP1 interactors. (**A**) Without SMURF2 enrichment. (**B**) After enforced expression of SMURF2WT. (**C**) In cells expressing catalytically-deficient SMURF2CG.

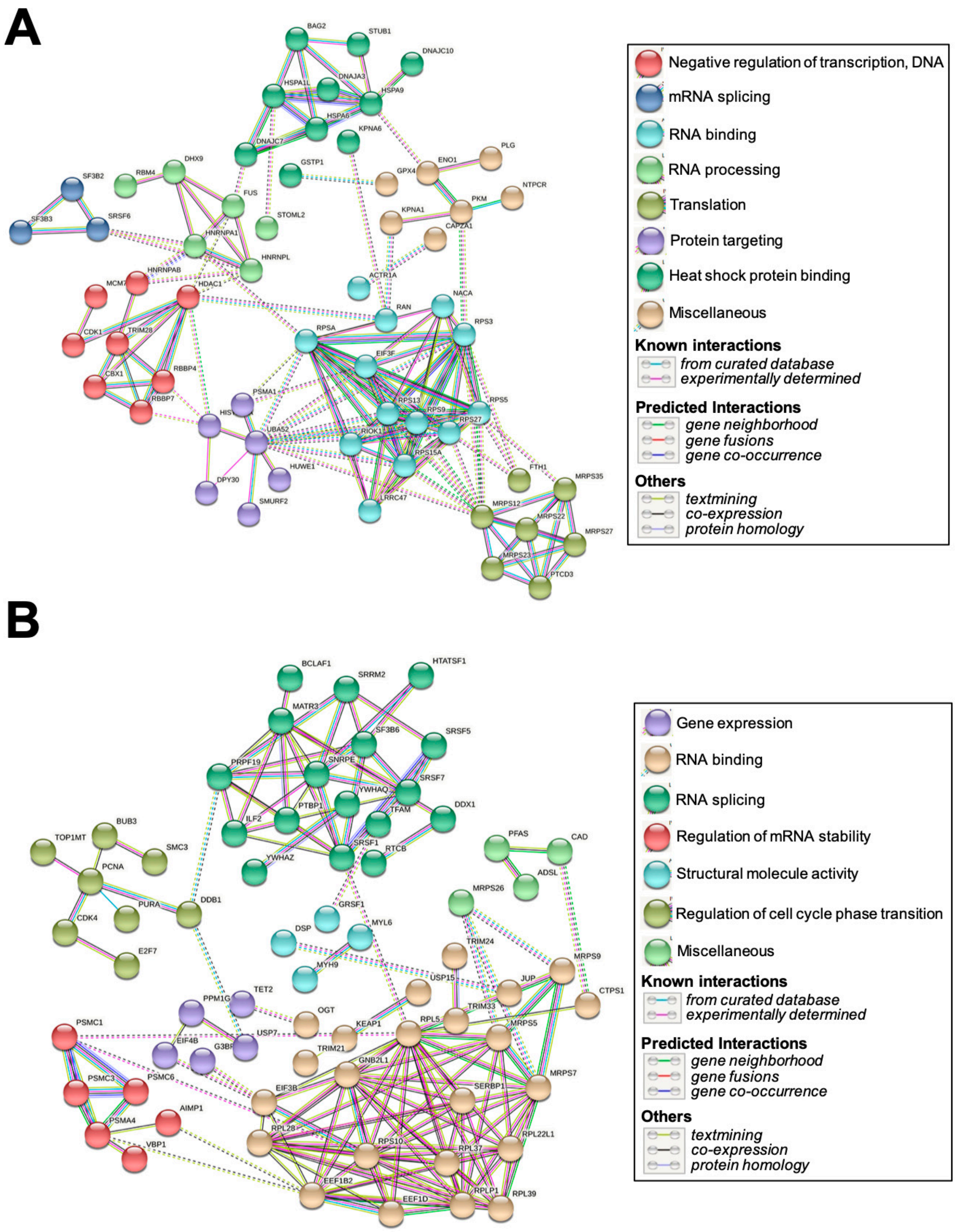

Figure S5. STRING analysis of protein-protein interaction network of KAP1 interactors affected by SMURF2. (**A**) Upregulated by SMURF2 (both WT and CG). (**B**) Downregulated by SMURF2 (both WT and CG). Only the proteins connected within the network are shown. Proteins are indicated by nodes labelled with the encoding gene symbol. Clusters identified by k-means clustering are shown in different colors.

Raw WB data for Fig. 1B

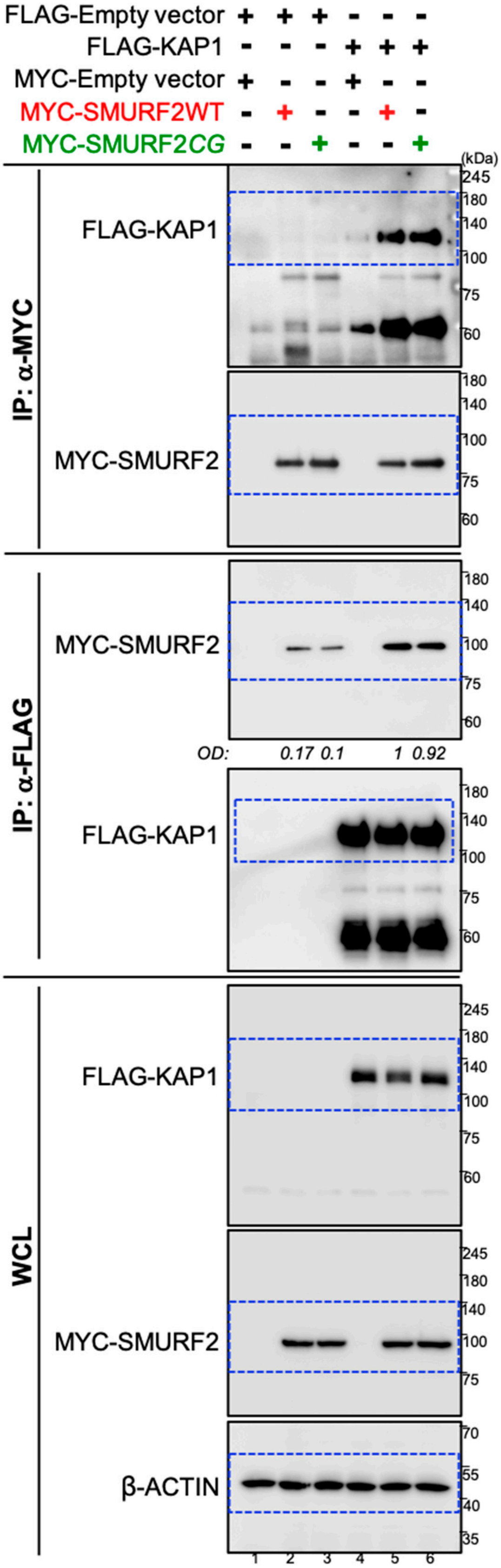

Raw WB data for Fig. 1C

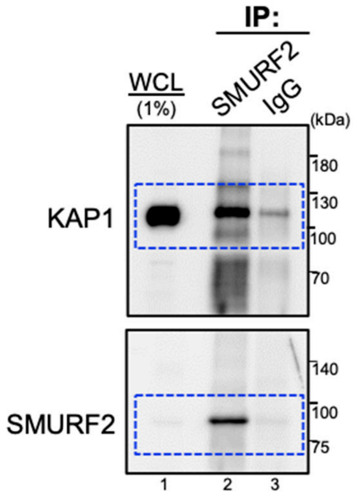

Raw WB data for Fig. 1G

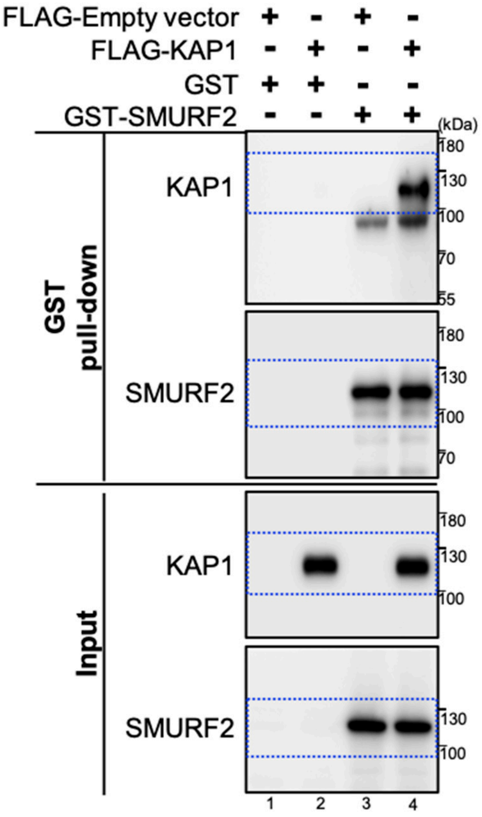

Raw WB data for Fig. 2A

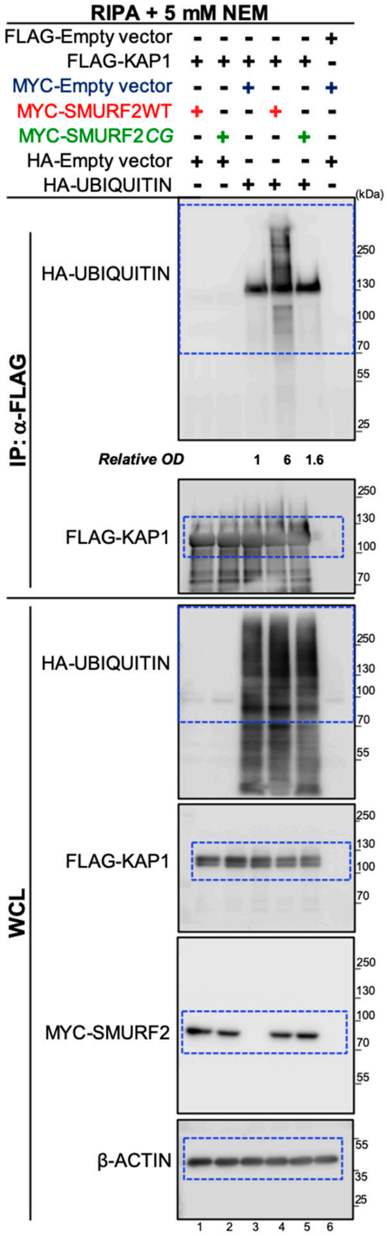

Raw WB data for Fig. 2C

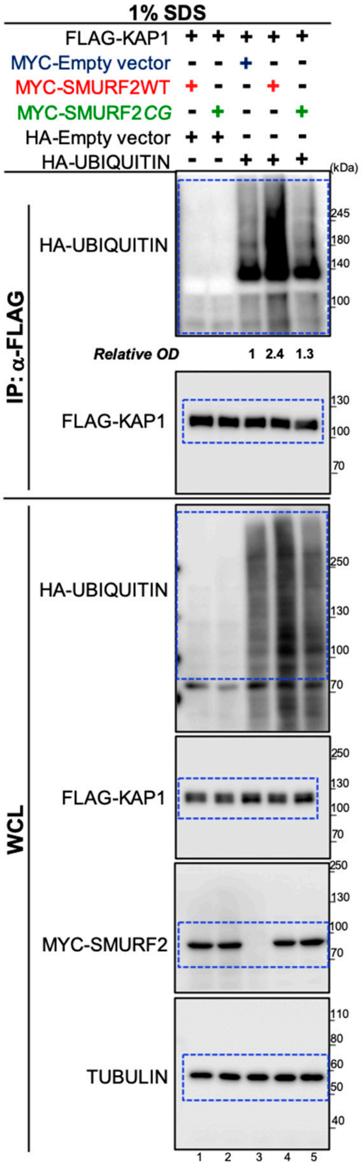

Raw WB data for Fig. 2E

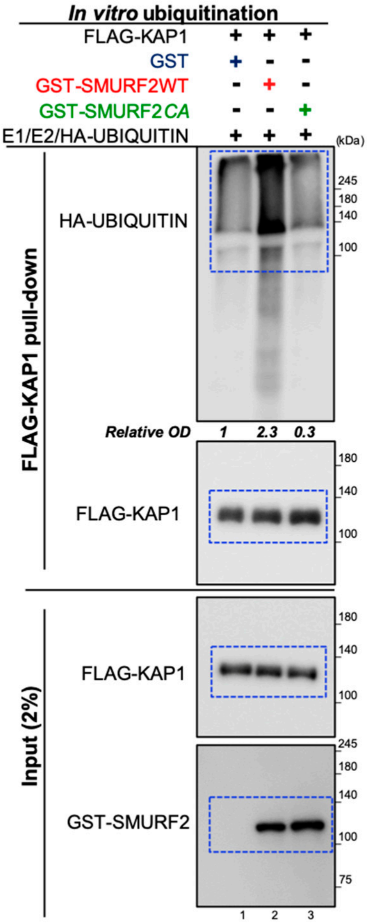

Raw WB data for Fig. 3A

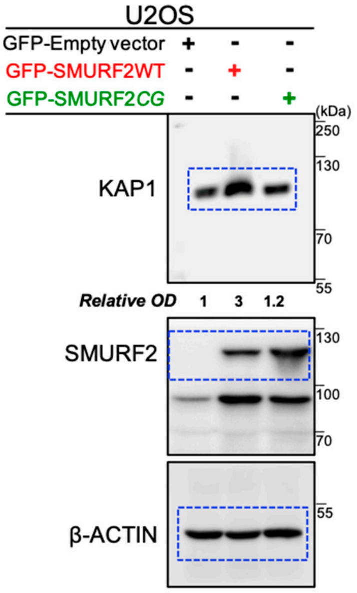

Raw WB data for Fig. 3B

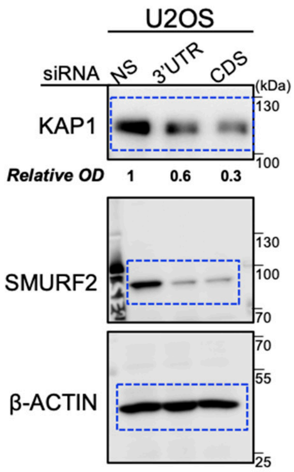

Raw WB data for Fig. 3C

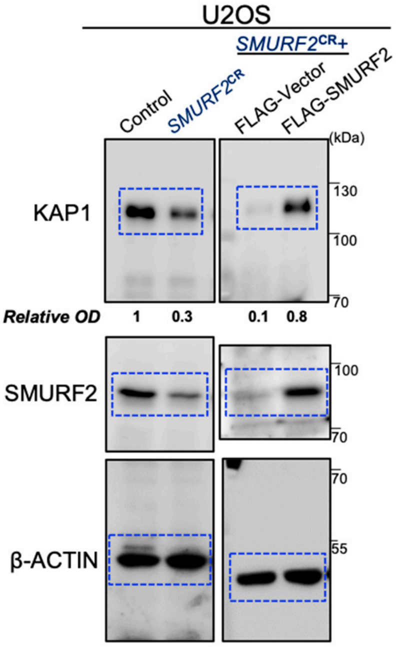

Raw WB data for Fig. 3D

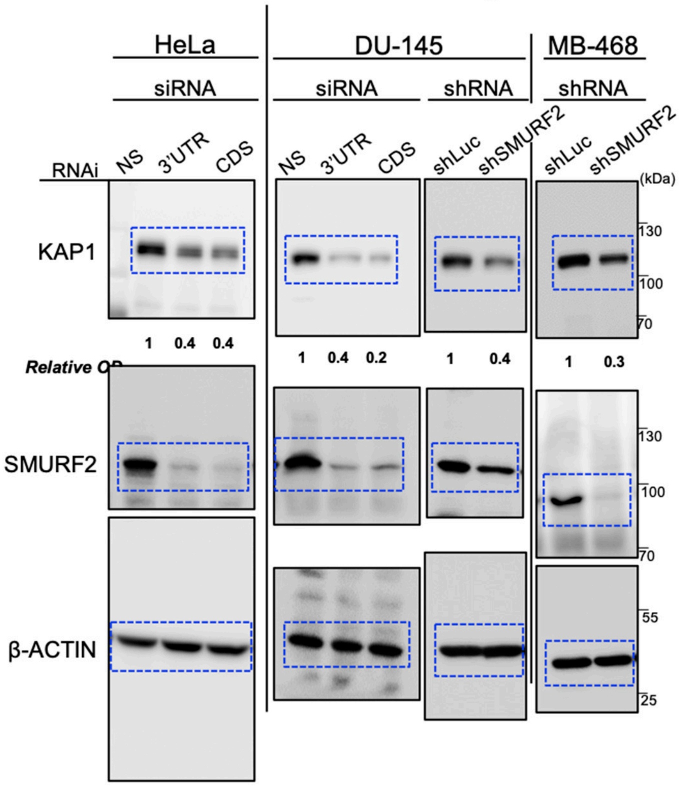

Raw WB data for Fig. 3G

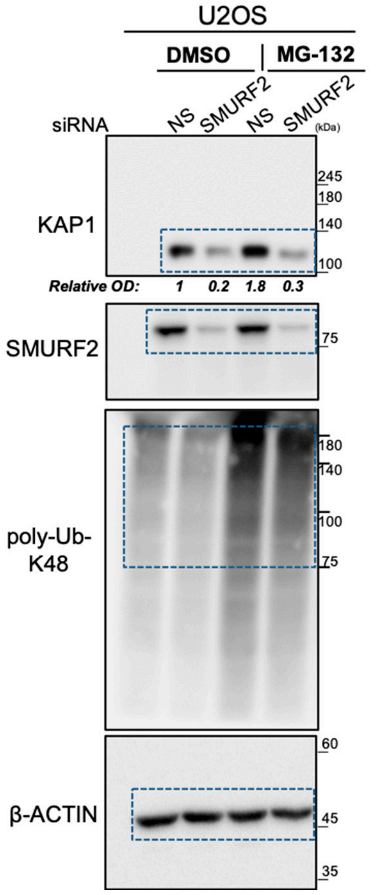

Raw WB data for Fig. 4A

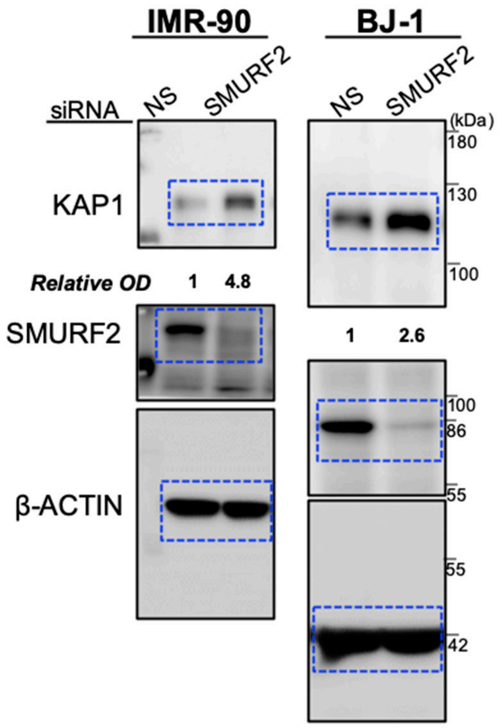

Raw WB data for Fig. 4B

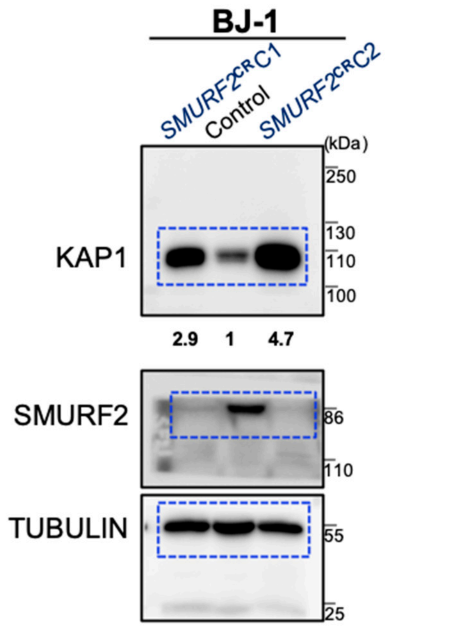

Raw WB data for Fig. 4E

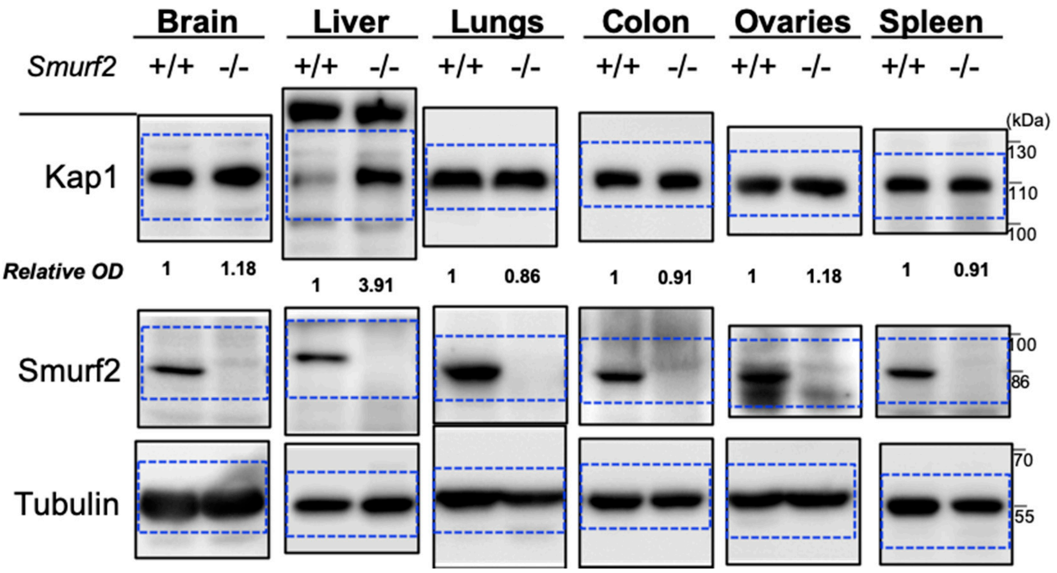

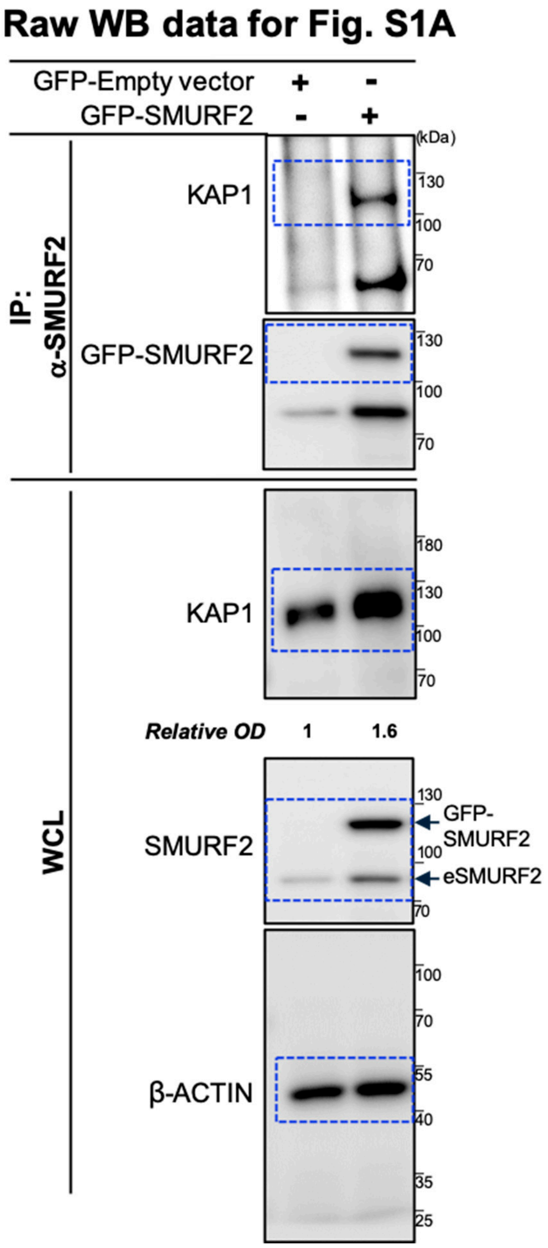
Raw WB data for Fig. S1A
GFP-Empty vector + -
GFP-SMURF2 - +
IP: α-SMURF2
KAP1
GFP-SMURF2
WCL
KAP1
Relative OD 1 1.6
SMURF2
GFP-SMURF2
eSMURF2
β-ACTIN
(kDa)
130
100
70
180
55
40
35
25

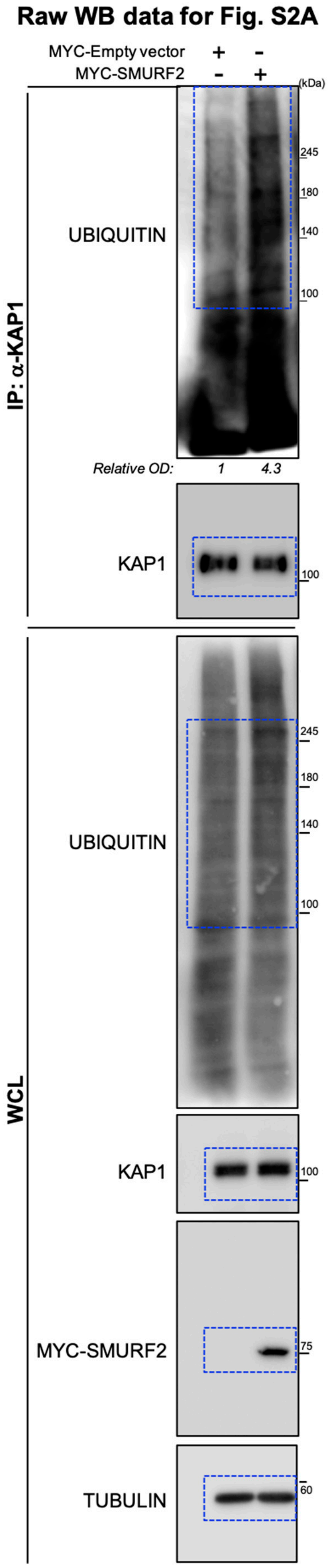

Figure S6. Uncropped Western Blot (WB) images.

Table S1. siRNA sequences.

siRNA	Sequence
Non-silencing (NS)	5′-CGUUAAUCGCGUAUAAUACGCGUAT-3′
SMURF2 #1 (3′UTR)	5′-UUCAGCAUAUUCUUUGAAACUCUGCUG-3′
SMURF2 #2 (CDS)	5′-AUAGGAUAAAUGUUCCGGAUUAACUGC-3′

Table S2. Primary antibodies used for immunoblotting.

Antibody	Dilution	Catalog Number (#) and Supplier
anti-KAP1	1:10000	#A300-274A, Bethyl
anti-SMURF2	1:1500	#12024, Cell Signaling
anti-FLAG	1:2000 to 1:4000	#F3165, Sigma
anti-MYC	1:1000	#2278, Cell Signaling
anti-HA	1:1000	#71-5500, Invitrogen/ThermoFisher
anti-β-Actin	1:3000	#600-401-886, Rockland
anti-α-Tubulin	1:4000	#T9026, Sigma
anti-Ubiquitin (P37)	1:1000	#58395, Cell Signaling
anti-K48-linkage specific polyubiquitin	1:2000	#8081, Cell Signaling

Table S3. qRT-PCR primers used in the study.

Gene	Primers	Location	Amplicon Size (bp)
KAP1	Forward: 5′-tgagacctgtgtagaggcg-3′	Exon 3	73
	Reverse: 5′-cgttcaccatcccgagactt-3′	Exon 4	
GAPDH	Forward: 5′-ggagcgagatccctccaaaat-3′	Exon 5	197
	Reverse: 5′-ggctgttgtcatacttctcatgg-3′	Exon 6	

Table S4. Details of GO terms for KAP1-interacting proteins changed in abundance by SMURF2. The data are sorted based on q-value FDR B&H.

Category	ID	Name	q-value FDR B&H	Count In Query List
**KAP1-interacting proteins *increased* in the presence of SMURF2 (wild-type and mutant forms)**				
**Molecular Functions**				
GO: Molecular Function	GO:0003723	RNA binding	7.55E−15	41
GO: Molecular Function	GO:0003735	structural constituent of ribosome	5.13E−08	13
GO: Molecular Function	GO:0031072	heat shock protein binding	5.75E−08	10
GO: Molecular Function	GO:0030544	Hsp70 protein binding	1.36E−04	5
GO: Molecular Function	GO:0003729	mRNA binding	6.55E−04	8
GO: Molecular Function	GO:0019843	rRNA binding	2.71E−03	5
GO: Molecular Function	GO:0005198	structural molecule activity	5.44E−03	14
GO: Molecular Function	GO:0051082	unfolded protein binding	1.57E−02	5
GO: Molecular Function	GO:0036002	pre-mRNA binding	1.78E−02	3
GO: Molecular Function	GO:0060590	ATPase regulator activity	3.19E−02	3
GO: Molecular Function	GO:0070181	small ribosomal subunit rRNA binding	3.19E−02	2
GO: Molecular Function	GO:0097157	pre-mRNA intronic binding	3.19E−02	2
GO: Molecular Function	GO:0051059	NF-kappaB binding	3.38E−02	3
**Biological Processes**				
GO: Biological Process	GO:0006412	translation	3.16E−09	23
GO: Biological Process	GO:0043043	peptide biosynthetic process	3.16E−09	23
GO: Biological Process	GO:0006518	peptide metabolic process	3.16E−09	25

GO: Biological Process	GO:0043604	amide biosynthetic process	3.16E−09	24
GO: Biological Process	GO:0043603	cellular amide metabolic process	2.01E−08	26
GO: Biological Process	GO:1901566	organonitrogen compound biosynthetic process	1.26E−06	28
GO: Biological Process	GO:0006413	translational initiation	3.52E−06	11
GO: Biological Process	GO:0006614	SRP-dependent cotranslational protein targeting to membrane	1.47E−05	8
GO: Biological Process	GO:0019080	viral gene expression	2.03E−05	10
GO: Biological Process	GO:0006613	cotranslational protein targeting to membrane	2.03E−05	8
GO: Biological Process	GO:0045047	protein targeting to ER	2.03E−05	8
GO: Biological Process	GO:0072599	establishment of protein localization to endoplasmic reticulum	2.51E−05	8
GO: Biological Process	GO:0016071	mRNA metabolic process	2.53E−05	17
GO: Biological Process	GO:0042254	ribosome biogenesis	3.54E−05	12
GO: Biological Process	GO:0044033	multi-organism metabolic process	3.54E−05	10
GO: Biological Process	GO:0022613	ribonucleoprotein complex biogenesis	4.53E−05	14
GO: Biological Process	GO:0000184	nuclear-transcribed mRNA catabolic process, nonsense-mediated decay	4.93E−05	8
GO: Biological Process	GO:0070972	protein localization to endoplasmic reticulum	5.97E−05	8
GO: Biological Process	GO:0019083	viral transcription	6.86E−05	9
GO: Biological Process	GO:0006396	RNA processing	6.86E−05	19
GO: Biological Process	GO:0006414	translational elongation	8.70E−05	8
GO: Biological Process	GO:0016032	viral process	9.68E−05	17
GO: Biological Process	GO:0019058	viral life cycle	1.14E−04	13
GO: Biological Process	GO:0044764	multi-organism cellular process	1.14E−04	17
GO: Biological Process	GO:0006364	rRNA processing	1.70E−04	10
GO: Biological Process	GO:0044403	symbiont process	1.85E−04	17
GO: Biological Process	GO:0044419	interspecies interaction between organisms	1.85E−04	17
GO: Biological Process	GO:0010608	posttranscriptional regulation of gene expression	1.86E−04	13
GO: Biological Process	GO:0016072	rRNA metabolic process	1.86E−04	10
GO: Biological Process	GO:0032543	mitochondrial translation	3.56E−04	7
GO: Biological Process	GO:0072594	establishment of protein localization to organelle	3.59E−04	15
GO: Biological Process	GO:0006457	protein folding	4.71E−04	9
GO: Biological Process	GO:0044265	cellular macromolecule catabolic process	4.71E−04	18
GO: Biological Process	GO:0070125	mitochondrial translational elongation	5.14E−04	6
GO: Biological Process	GO:0070126	mitochondrial translational termination	5.34E−04	6
GO: Biological Process	GO:0006605	protein targeting	6.89E−04	15
GO: Biological Process	GO:0006612	protein targeting to membrane	7.74E−04	8
GO: Biological Process	GO:0000956	nuclear-transcribed mRNA catabolic process	1.01E−03	8
GO: Biological Process	GO:0006415	translational termination	1.08E−03	6
GO: Biological Process	GO:0006417	regulation of translation	1.33E−03	10
GO: Biological Process	GO:0010629	negative regulation of gene expression	1.33E−03	23
GO: Biological Process	GO:0033119	negative regulation of RNA splicing	1.33E−03	4
GO: Biological Process	GO:0009890	negative regulation of biosynthetic process	1.40E−03	23
GO: Biological Process	GO:0051172	negative regulation of nitrogen compound metabolic process	1.44E−03	23
GO: Biological Process	GO:0006402	mRNA catabolic process	1.47E−03	8
GO: Biological Process	GO:0006259	DNA metabolic process	1.58E−03	17
GO: Biological Process	GO:0034605	cellular response to heat	1.89E−03	6
GO: Biological Process	GO:0009057	macromolecule catabolic process	1.91E−03	19
GO: Biological Process	GO:0033365	protein localization to organelle	2.13E−03	16
GO: Biological Process	GO:0009408	response to heat	2.13E−03	7
GO: Biological Process	GO:0034248	regulation of cellular amide metabolic process	2.18E−03	10
GO: Biological Process	GO:0000398	mRNA splicing, via spliceosome	2.18E−03	9
GO: Biological Process	GO:0000377	RNA splicing, via transesterification reactions with bulged adenosine as nucleophile	2.18E−03	9
GO: Biological Process	GO:0000375	RNA splicing, via transesterification reactions	2.36E−03	9
GO: Biological Process	GO:0031327	negative regulation of cellular biosynthetic process	2.51E−03	22

GO: Biological Process	GO:0006401	RNA catabolic process	2.86E−03	8
GO: Biological Process	GO:0045934	negative regulation of nucleobase-containing compound metabolic process	2.94E−03	21
GO: Biological Process	GO:0034470	ncRNA processing	2.97E−03	10
GO: Biological Process	GO:0022411	cellular component disassembly	3.02E−03	13
GO: Biological Process	GO:0008380	RNA splicing	3.06E−03	10
GO: Biological Process	GO:1901165	positive regulation of trophoblast cell migration	3.29E−03	2
GO: Biological Process	GO:0010558	negative regulation of macromolecule biosynthetic process	3.64E−03	21
GO: Biological Process	GO:0034655	nucleobase-containing compound catabolic process	4.02E−03	10
GO: Biological Process	GO:2000113	negative regulation of cellular macromolecule biosynthetic process	4.64E−03	20
GO: Biological Process	GO:0000028	ribosomal small subunit assembly	5.98E−03	3
GO: Biological Process	GO:0051253	negative regulation of RNA metabolic process	5.98E−03	19
GO: Biological Process	GO:0070370	cellular heat acclimation	5.98E−03	2
GO: Biological Process	GO:0043624	cellular protein complex disassembly	6.17E−03	7
GO: Biological Process	GO:0071103	DNA conformation change	6.22E−03	8
GO: Biological Process	GO:0044270	cellular nitrogen compound catabolic process	8.11E−03	10
GO: Biological Process	GO:0046700	heterocycle catabolic process	8.28E−03	10
GO: Biological Process	GO:0042026	protein refolding	8.35E−03	3
GO: Biological Process	GO:0042255	ribosome assembly	8.61E−03	4
GO: Biological Process	GO:0010286	heat acclimation	8.92E−03	2
GO: Biological Process	GO:0034660	ncRNA metabolic process	8.92E−03	11
GO: Biological Process	GO:0045727	positive regulation of translation	9.04E−03	5
GO: Biological Process	GO:0019439	aromatic compound catabolic process	9.04E−03	10
GO: Biological Process	GO:0007005	mitochondrion organization	9.54E−03	13
GO: Biological Process	GO:0009266	response to temperature stimulus	9.89E−03	7
GO: Biological Process	GO:0043484	regulation of RNA splicing	9.89E−03	5
GO: Biological Process	GO:0048025	negative regulation of mRNA splicing, via spliceosome	1.07E−02	3
GO: Biological Process	GO:0006886	intracellular protein transport	1.07E−02	16
GO: Biological Process	GO:0006913	nucleocytoplasmic transport	1.16E−02	10
GO: Biological Process	GO:0030579	ubiquitin-dependent SMAD protein catabolic process	1.18E−02	2
GO: Biological Process	GO:0051169	nuclear transport	1.29E−02	10
GO: Biological Process	GO:1901361	organic cyclic compound catabolic process	1.44E−02	10
GO: Biological Process	GO:0071389	cellular response to mineralocorticoid stimulus	1.58E−02	2
GO: Biological Process	GO:1901163	regulation of trophoblast cell migration	1.58E−02	2
GO: Biological Process	GO:0051168	nuclear export	1.60E−02	6
GO: Biological Process	GO:0048024	regulation of mRNA splicing, via spliceosome	1.62E−02	4
GO: Biological Process	GO:0034250	positive regulation of cellular amide metabolic process	1.63E−02	5
GO: Biological Process	GO:0048147	negative regulation of fibroblast proliferation	1.76E−02	3
GO: Biological Process	GO:0043044	ATP-dependent chromatin remodeling	1.93E−02	4
GO: Biological Process	GO:0061450	trophoblast cell migration	1.96E−02	2
GO: Biological Process	GO:0032984	protein-containing complex disassembly	2.17E−02	7
GO: Biological Process	GO:0000381	regulation of alternative mRNA splicing, via spliceosome	2.37E−02	3
GO: Biological Process	GO:0042769	DNA damage response, detection of DNA damage	2.37E−02	3
GO: Biological Process	GO:0031943	regulation of glucocorticoid metabolic process	2.41E−02	2
GO: Biological Process	GO:0022618	ribonucleoprotein complex assembly	2.43E−02	6
GO: Biological Process	GO:0050686	negative regulation of mRNA processing	2.49E−02	3
GO: Biological Process	GO:0034622	cellular protein-containing complex assembly	2.61E−02	14
GO: Biological Process	GO:0090150	establishment of protein localization to membrane	2.66E−02	8
GO: Biological Process	GO:0071826	ribonucleoprotein complex subunit organization	2.97E−02	6
GO: Biological Process	GO:0006397	mRNA processing	3.03E−02	9
GO: Biological Process	GO:0045892	negative regulation of transcription, DNA-templated	3.37E−02	16
GO: Biological Process	GO:0061641	CENP-A containing chromatin organization	3.37E−02	3
GO: Biological Process	GO:0034080	CENP-A containing nucleosome assembly	3.37E−02	3

GO: Biological Process	GO:1901575	organic substance catabolic process	3.66E−02	21
GO: Biological Process	GO:1903312	negative regulation of mRNA metabolic process	3.79E−02	3
GO: Biological Process	GO:0002192	IRES-dependent translational initiation of linear mRNA	3.85E−02	2
GO: Biological Process	GO:0071428	rRNA-containing ribonucleoprotein complex export from nucleus	3.85E−02	2
GO: Biological Process	GO:0031055	chromatin remodeling at centromere	4.19E−02	3
GO: Biological Process	GO:0000380	alternative mRNA splicing, via spliceosome	4.39E−02	3
GO: Biological Process	GO:1903507	negative regulation of nucleic acid-templated transcription	4.39E−02	16
GO: Biological Process	GO:0072657	protein localization to membrane	4.48E−02	9
GO: Biological Process	GO:0006754	ATP biosynthetic process	4.57E−02	3
GO: Biological Process	GO:0051276	chromosome organization	4.57E−02	15
GO: Biological Process	GO:0044248	cellular catabolic process	4.63E−02	20
GO: Biological Process	GO:1902679	negative regulation of RNA biosynthetic process	4.71E−02	16
GO: Biological Process	GO:0051443	positive regulation of ubiquitin-protein transferase activity	4.82E−02	4
GO: Biological Process	GO:2000674	regulation of type B pancreatic cell apoptotic process	4.84E−02	2
		**Cellular Components**		
GO: Cellular Component	GO:0015935	small ribosomal subunit	5.53E−11	11
GO: Cellular Component	GO:0030529	intracellular ribonucleoprotein complex	1.47E−10	24
GO: Cellular Component	GO:1990904	ribonucleoprotein complex	1.47E−10	24
GO: Cellular Component	GO:0044391	ribosomal subunit	1.66E−08	12
GO: Cellular Component	GO:0005840	ribosome	3.22E−08	13
GO: Cellular Component	GO:0022627	cytosolic small ribosomal subunit	2.06E−07	7
GO: Cellular Component	GO:0022626	cytosolic ribosome	1.32E−05	8
GO: Cellular Component	GO:0044445	cytosolic part	2.58E−05	10
GO: Cellular Component	GO:0019866	organelle inner membrane	4.37E−04	13
GO: Cellular Component	GO:0005763	mitochondrial small ribosomal subunit	4.57E−04	4
GO: Cellular Component	GO:0000314	organellar small ribosomal subunit	4.57E−04	4
GO: Cellular Component	GO:0005743	mitochondrial inner membrane	5.44E−04	12
GO: Cellular Component	GO:0005739	mitochondrion	5.44E−04	24
GO: Cellular Component	GO:0044429	mitochondrial part	5.44E−04	17
GO: Cellular Component	GO:0005759	mitochondrial matrix	2.39E−03	10
GO: Cellular Component	GO:0043209	myelin sheath	2.45E−03	7
GO: Cellular Component	GO:0016581	NuRD complex	2.59E−03	3
GO: Cellular Component	GO:0090545	CHD-type complex	2.59E−03	3
GO: Cellular Component	GO:0005740	mitochondrial envelope	3.06E−03	13
GO: Cellular Component	GO:0031967	organelle envelope	3.06E−03	17
GO: Cellular Component	GO:0031975	envelope	3.07E−03	17
GO: Cellular Component	GO:0000781	chromosome, telomeric region	3.88E−03	6
GO: Cellular Component	GO:0090568	nuclear transcriptional repressor complex	5.29E−03	3
GO: Cellular Component	GO:0031966	mitochondrial membrane	5.40E−03	12
GO: Cellular Component	GO:0000784	nuclear chromosome, telomeric region	1.04E−02	5
GO: Cellular Component	GO:0008180	COP9 signalosome	1.04E−02	3
GO: Cellular Component	GO:0000313	organellar ribosome	1.07E−02	4
GO: Cellular Component	GO:0005761	mitochondrial ribosome	1.07E−02	4
GO: Cellular Component	GO:0005730	nucleolus	1.25E−02	13
GO: Cellular Component	GO:0005844	polysome	2.63E−02	3
GO: Cellular Component	GO:0016580	Sin3 complex	2.70E−02	2
GO: Cellular Component	GO:0035098	ESC/E(Z) complex	3.42E−02	2
GO: Cellular Component	GO:0070822	Sin3-type complex	3.74E−02	2
GO: Cellular Component	GO:0000118	histone deacetylase complex	4.72E−02	3
		**KAP1-interacting proteins *decreased* in the presence of SMURF2 (wild-type and mutant forms)**		
		**Molecular Functions**		
GO: Molecular Function	GO:0003723	RNA binding	4.35E−20	56
GO: Molecular Function	GO:0003735	structural constituent of ribosome	2.87E−05	12
GO: Molecular Function	GO:0005198	structural molecule activity	2.95E−04	20

GO: Molecular Function	GO:0036402	proteasome-activating ATPase activity	9.85E−04	3
GO: Molecular Function	GO:0003729	mRNA binding	1.01E−03	9
GO: Molecular Function	GO:0042623	ATPase activity, coupled	4.41E−03	11
GO: Molecular Function	GO:0016887	ATPase activity	1.25E−02	12
GO: Molecular Function	GO:0016879	ligase activity, forming carbon-nitrogen bonds	2.71E−02	9
GO: Molecular Function	GO:0017025	TBP-class protein binding	3.47E−02	3
GO: Molecular Function	GO:0003700	DNA binding transcription factor activity	3.47E−02	21
GO: Molecular Function	GO:0086083	cell adhesive protein binding involved in bundle of His cell-Purkinje myocyte communication	3.47E−02	2
GO: Molecular Function	GO:0050733	RS domain binding	4.43E−02	2
		**Biological Processes**		
GO: Biological Process	GO:0006412	translation	1.57E−07	25
GO: Biological Process	GO:0043043	peptide biosynthetic process	1.92E−07	25
GO: Biological Process	GO:0043604	amide biosynthetic process	2.01E−07	26
GO: Biological Process	GO:0016071	mRNA metabolic process	2.01E−07	24
GO: Biological Process	GO:0006396	RNA processing	2.01E−07	28
GO: Biological Process	GO:0008380	RNA splicing	9.75E−07	18
GO: Biological Process	GO:0006518	peptide metabolic process	3.02E−06	25
GO: Biological Process	GO:0043603	cellular amide metabolic process	2.63E−05	26
GO: Biological Process	GO:1901566	organonitrogen compound biosynthetic process	4.84E−05	31
GO: Biological Process	GO:0006397	mRNA processing	4.84E−05	17
GO: Biological Process	GO:0000398	mRNA splicing, via spliceosome	1.54E−04	13
GO: Biological Process	GO:0000377	RNA splicing, via transesterification reactions with bulged adenosine as nucleophile	1.54E−04	13
GO: Biological Process	GO:0000375	RNA splicing, via transesterification reactions	1.60E−04	13
GO: Biological Process	GO:0010608	posttranscriptional regulation of gene expression	1.60E−04	16
GO: Biological Process	GO:0044265	cellular macromolecule catabolic process	3.15E−04	23
GO: Biological Process	GO:0006413	translational initiation	4.19E−04	10
GO: Biological Process	GO:0022613	ribonucleoprotein complex biogenesis	5.36E−04	15
GO: Biological Process	GO:0022618	ribonucleoprotein complex assembly	6.93E−04	10
GO: Biological Process	GO:0071826	ribonucleoprotein complex subunit organization	9.92E−04	10
GO: Biological Process	GO:0019080	viral gene expression	1.77E−03	9
GO: Biological Process	GO:1903321	negative regulation of protein modification by small protein conjugation or removal	1.77E−03	8
GO: Biological Process	GO:0009057	macromolecule catabolic process	2.65E−03	24
GO: Biological Process	GO:0016032	viral process	2.65E−03	18
GO: Biological Process	GO:0044764	multi-organism cellular process	3.22E−03	18
GO: Biological Process	GO:0044033	multi-organism metabolic process	3.28E−03	9
GO: Biological Process	GO:2000144	positive regulation of DNA-templated transcription, initiation	3.95E−03	4
GO: Biological Process	GO:0070647	protein modification by small protein conjugation or removal	4.01E−03	21
GO: Biological Process	GO:0019058	viral life cycle	4.01E−03	13
GO: Biological Process	GO:0070972	protein localization to endoplasmic reticulum	4.34E−03	7
GO: Biological Process	GO:0044403	symbiont process	4.35E−03	18
GO: Biological Process	GO:0044419	interspecies interaction between organisms	4.35E−03	18
GO: Biological Process	GO:0002181	cytoplasmic translation	4.35E−03	5
GO: Biological Process	GO:0065003	protein-containing complex assembly	4.58E−03	30
GO: Biological Process	GO:1901987	regulation of cell cycle phase transition	4.72E−03	11
GO: Biological Process	GO:0000245	spliceosomal complex assembly	5.01E−03	5
GO: Biological Process	GO:0031124	mRNA 3'-end processing	5.01E−03	6
GO: Biological Process	GO:0031397	negative regulation of protein ubiquitination	5.01E−03	7
GO: Biological Process	GO:1903052	positive regulation of proteolysis involved in cellular protein catabolic process	5.01E−03	8
GO: Biological Process	GO:0006414	translational elongation	5.01E−03	7
GO: Biological Process	GO:0034622	cellular protein-containing complex assembly	5.09E−03	20

GO: Biological Process	GO:0006614	SRP-dependent cotranslational protein targeting to membrane	5.09E−03	6
GO: Biological Process	GO:0045899	positive regulation of RNA polymerase II transcriptional preinitiation complex assembly	5.09E−03	3
GO: Biological Process	GO:1901800	positive regulation of proteasomal protein catabolic process	5.80E−03	6
GO: Biological Process	GO:2000142	regulation of DNA-templated transcription, initiation	6.01E−03	4
GO: Biological Process	GO:0006369	termination of RNA polymerase II transcription	6.01E−03	5
GO: Biological Process	GO:0043488	regulation of mRNA stability	6.64E−03	7
GO: Biological Process	GO:0006613	cotranslational protein targeting to membrane	6.64E−03	6
GO: Biological Process	GO:1903364	positive regulation of cellular protein catabolic process	6.70E−03	8
GO: Biological Process	GO:0045047	protein targeting to ER	6.72E−03	6
GO: Biological Process	GO:2000058	regulation of ubiquitin-dependent protein catabolic process	6.95E−03	6
GO: Biological Process	GO:0043487	regulation of RNA stability	7.12E−03	7
GO: Biological Process	GO:0042769	DNA damage response, detection of DNA damage	7.32E−03	4
GO: Biological Process	GO:0072599	establishment of protein localization to endoplasmic reticulum	7.57E−03	6
GO: Biological Process	GO:0051028	mRNA transport	7.57E−03	7
GO: Biological Process	GO:0090150	establishment of protein localization to membrane	7.57E−03	11
GO: Biological Process	GO:0000956	nuclear-transcribed mRNA catabolic process	7.57E−03	8
GO: Biological Process	GO:0045898	regulation of RNA polymerase II transcriptional preinitiation complex assembly	7.62E−03	3
GO: Biological Process	GO:1901990	regulation of mitotic cell cycle phase transition	7.80E−03	10
GO: Biological Process	GO:0051351	positive regulation of ligase activity	8.02E−03	6
GO: Biological Process	GO:0051603	proteolysis involved in cellular protein catabolic process	8.06E−03	15
GO: Biological Process	GO:0006511	ubiquitin-dependent protein catabolic process	8.10E−03	14
GO: Biological Process	GO:0032446	protein modification by small protein conjugation	8.85E−03	18
GO: Biological Process	GO:1903050	regulation of proteolysis involved in cellular protein catabolic process	8.86E−03	9
GO: Biological Process	GO:0019941	modification-dependent protein catabolic process	8.86E−03	14
GO: Biological Process	GO:1904667	negative regulation of ubiquitin protein ligase activity	9.02E−03	5
GO: Biological Process	GO:0044270	cellular nitrogen compound catabolic process	9.36E−03	12
GO: Biological Process	GO:0006403	RNA localization	9.36E−03	8
GO: Biological Process	GO:0043632	modification-dependent macromolecule catabolic process	9.42E−03	14
GO: Biological Process	GO:0046700	heterocycle catabolic process	9.42E−03	12
GO: Biological Process	GO:0034470	ncRNA processing	9.72E−03	11
GO: Biological Process	GO:0006402	mRNA catabolic process	1.00E−02	8
GO: Biological Process	GO:0006520	cellular amino acid metabolic process	1.01E−02	11
GO: Biological Process	GO:0031145	anaphase-promoting complex-dependent catabolic process	1.03E−02	5
GO: Biological Process	GO:0046726	positive regulation by virus of viral protein levels in host cell	1.04E−02	2
GO: Biological Process	GO:0031123	RNA 3'-end processing	1.04E−02	6
GO: Biological Process	GO:0044257	cellular protein catabolic process	1.04E−02	15
GO: Biological Process	GO:1901575	organic substance catabolic process	1.04E−02	29
GO: Biological Process	GO:0000184	nuclear-transcribed mRNA catabolic process, nonsense-mediated decay	1.07E−02	6
GO: Biological Process	GO:0044248	cellular catabolic process	1.12E−02	28
GO: Biological Process	GO:0060261	positive regulation of transcription initiation from RNA polymerase II promoter	1.12E−02	3
GO: Biological Process	GO:0051340	regulation of ligase activity	1.12E−02	6
GO: Biological Process	GO:0016567	protein ubiquitination	1.12E−02	16
GO: Biological Process	GO:0044770	cell cycle phase transition	1.12E−02	13
GO: Biological Process	GO:1904668	positive regulation of ubiquitin protein ligase activity	1.12E−02	5
GO: Biological Process	GO:0051444	negative regulation of ubiquitin-protein transferase activity	1.12E−02	5
GO: Biological Process	GO:1903362	regulation of cellular protein catabolic process	1.12E−02	9
GO: Biological Process	GO:0034655	nucleobase-containing compound catabolic process	1.16E−02	11
GO: Biological Process	GO:1903320	regulation of protein modification by small protein conjugation or removal	1.17E−02	9
GO: Biological Process	GO:0019083	viral transcription	1.17E−02	7

GO: Biological Process	GO:0098901	regulation of cardiac muscle cell action potential	1.17E−02	3
GO: Biological Process	GO:0051352	negative regulation of ligase activity	1.17E−02	5
GO: Biological Process	GO:0010498	proteasomal protein catabolic process	1.18E−02	11
GO: Biological Process	GO:0050658	RNA transport	1.23E−02	7
GO: Biological Process	GO:0050657	nucleic acid transport	1.23E−02	7
GO: Biological Process	GO:1904666	regulation of ubiquitin protein ligase activity	1.25E−02	5
GO: Biological Process	GO:0051236	establishment of RNA localization	1.33E−02	7
GO: Biological Process	GO:1901361	organic cyclic compound catabolic process	1.55E−02	12
GO: Biological Process	GO:0061136	regulation of proteasomal protein catabolic process	1.62E−02	7
GO: Biological Process	GO:0006401	RNA catabolic process	1.64E−02	8
GO: Biological Process	GO:2000060	positive regulation of ubiquitin-dependent protein catabolic process	1.70E−02	5
GO: Biological Process	GO:0051123	RNA polymerase II transcriptional preinitiation complex assembly	1.81E−02	3
GO: Biological Process	GO:0006612	protein targeting to membrane	1.81E−02	7
GO: Biological Process	GO:0045862	positive regulation of proteolysis	1.81E−02	10
GO: Biological Process	GO:0000209	protein polyubiquitination	1.83E−02	8
GO: Biological Process	GO:0006189	'de novo' IMP biosynthetic process	1.83E−02	2
GO: Biological Process	GO:0086073	bundle of His cell-Purkinje myocyte adhesion involved in cell communication	1.83E−02	2
GO: Biological Process	GO:0051726	regulation of cell cycle	1.87E−02	18
GO: Biological Process	GO:0006541	glutamine metabolic process	1.90E−02	3
GO: Biological Process	GO:0060260	regulation of transcription initiation from RNA polymerase II promoter	1.90E−02	3
GO: Biological Process	GO:0030163	protein catabolic process	1.91E−02	16
GO: Biological Process	GO:0070897	DNA-templated transcriptional preinitiation complex assembly	2.10E−02	3
GO: Biological Process	GO:0043161	proteasome-mediated ubiquitin-dependent protein catabolic process	2.10E−02	10
GO: Biological Process	GO:0007049	cell cycle	2.32E−02	26
GO: Biological Process	GO:0031396	regulation of protein ubiquitination	2.32E−02	8
GO: Biological Process	GO:0075522	IRES-dependent viral translational initiation	2.32E−02	2
GO: Biological Process	GO:0086042	cardiac muscle cell-cardiac muscle cell adhesion	2.32E−02	2
GO: Biological Process	GO:0006353	DNA-templated transcription, termination	2.32E−02	5
GO: Biological Process	GO:0045732	positive regulation of protein catabolic process	2.32E−02	8
GO: Biological Process	GO:0019439	aromatic compound catabolic process	2.36E−02	11
GO: Biological Process	GO:0051443	positive regulation of ubiquitin-protein transferase activity	2.36E−02	5
GO: Biological Process	GO:0043624	cellular protein complex disassembly	2.62E−02	7
GO: Biological Process	GO:0071427	mRNA-containing ribonucleoprotein complex export from nucleus	2.62E−02	5
GO: Biological Process	GO:0006406	mRNA export from nucleus	2.62E−02	5
GO: Biological Process	GO:0015931	nucleobase-containing compound transport	2.71E−02	7
GO: Biological Process	GO:0002479	antigen processing and presentation of exogenous peptide antigen via MHC class I, TAP-dependent	2.77E−02	4
GO: Biological Process	GO:0034660	ncRNA metabolic process	2.79E−02	12
GO: Biological Process	GO:0032875	regulation of DNA endoreduplication	2.83E−02	2
GO: Biological Process	GO:0006417	regulation of translation	2.86E−02	9
GO: Biological Process	GO:0006521	regulation of cellular amino acid metabolic process	3.01E−02	4
GO: Biological Process	GO:0086004	regulation of cardiac muscle cell contraction	3.01E−02	3
GO: Biological Process	GO:0031331	positive regulation of cellular catabolic process	3.06E−02	9
GO: Biological Process	GO:0071103	DNA conformation change	3.06E−02	8
GO: Biological Process	GO:0042590	antigen processing and presentation of exogenous peptide antigen via MHC class I	3.26E−02	4
GO: Biological Process	GO:0051172	negative regulation of nitrogen compound metabolic process	3.37E−02	24
GO: Biological Process	GO:0042023	DNA endoreduplication	3.40E−02	2
GO: Biological Process	GO:0030433	ubiquitin-dependent ERAD pathway	3.54E−02	4

GO: Biological Process	GO:0007346	regulation of mitotic cell cycle	3.54E−02	11
GO: Biological Process	GO:0009064	glutamine family amino acid metabolic process	3.66E−02	4
GO: Biological Process	GO:0044786	cell cycle DNA replication	3.66E−02	3
GO: Biological Process	GO:0046131	pyrimidine ribonucleoside metabolic process	3.66E−02	3
GO: Biological Process	GO:0072657	protein localization to membrane	3.82E−02	11
GO: Biological Process	GO:0051438	regulation of ubiquitin-protein transferase activity	3.85E−02	5
GO: Biological Process	GO:0071426	ribonucleoprotein complex export from nucleus	3.85E−02	5
GO: Biological Process	GO:0006405	RNA export from nucleus	3.85E−02	5
GO: Biological Process	GO:0006283	transcription-coupled nucleotide-excision repair	3.86E−02	4
GO: Biological Process	GO:0019081	viral translation	3.86E−02	2
GO: Biological Process	GO:0002934	desmosome organization	3.86E−02	2
GO: Biological Process	GO:1903047	mitotic cell cycle process	3.86E−02	16
GO: Biological Process	GO:0010564	regulation of cell cycle process	3.88E−02	12
GO: Biological Process	GO:0071166	ribonucleoprotein complex localization	4.11E−02	5
GO: Biological Process	GO:0010948	negative regulation of cell cycle process	4.14E−02	7
GO: Biological Process	GO:0090263	positive regulation of canonical Wnt signaling pathway	4.20E−02	5
GO: Biological Process	GO:0032508	DNA duplex unwinding	4.24E−02	4
GO: Biological Process	GO:1903115	regulation of actin filament-based movement	4.24E−02	3
GO: Biological Process	GO:0044772	mitotic cell cycle phase transition	4.24E−02	11
GO: Biological Process	GO:0030048	actin filament-based movement	4.24E−02	5
GO: Biological Process	GO:0034248	regulation of cellular amide metabolic process	4.29E−02	9
GO: Biological Process	GO:0098911	regulation of ventricular cardiac muscle cell action potential	4.29E−02	2
GO: Biological Process	GO:0086069	bundle of His cell to Purkinje myocyte communication	4.29E−02	2
GO: Biological Process	GO:0006188	IMP biosynthetic process	4.29E−02	2
GO: Biological Process	GO:0051014	actin filament severing	4.29E−02	2
GO: Biological Process	GO:0046719	regulation by virus of viral protein levels in host cell	4.29E−02	2
GO: Biological Process	GO:0072527	pyrimidine-containing compound metabolic process	4.80E−02	4
GO: Biological Process	GO:0051168	nuclear export	4.88E−02	6
GO: Biological Process	GO:0032392	DNA geometric change	4.94E−02	4
GO: Biological Process	GO:0031398	positive regulation of protein ubiquitination	4.94E−02	6
GO: Biological Process	GO:0002192	IRES-dependent translational initiation of linear mRNA	4.96E−02	2
		**Cellular Components**		
GO: Cellular Component	GO:0030529	intracellular ribonucleoprotein complex	1.50E−12	31
GO: Cellular Component	GO:1990904	ribonucleoprotein complex	1.50E−12	31
GO: Cellular Component	GO:0044391	ribosomal subunit	1.24E−06	12
GO: Cellular Component	GO:0005840	ribosome	2.38E−05	12
GO: Cellular Component	GO:0044445	cytosolic part	1.43E−04	11
GO: Cellular Component	GO:0022626	cytosolic ribosome	2.22E−04	8
GO: Cellular Component	GO:0022625	cytosolic large ribosomal subunit	6.00E−04	6
GO: Cellular Component	GO:0005913	cell-cell adherens junction	6.00E−04	6
GO: Cellular Component	GO:0015935	small ribosomal subunit	6.87E−04	6
GO: Cellular Component	GO:0005912	adherens junction	6.87E−04	14
GO: Cellular Component	GO:0035770	ribonucleoprotein granule	6.87E−04	8
GO: Cellular Component	GO:0031595	nuclear proteasome complex	6.87E−04	3
GO: Cellular Component	GO:0016607	nuclear speck	6.87E−04	9
GO: Cellular Component	GO:0070161	anchoring junction	8.43E−04	14
GO: Cellular Component	GO:0005681	spliceosomal complex	1.23E−03	8
GO: Cellular Component	GO:0005763	mitochondrial small ribosomal subunit	1.23E−03	4
GO: Cellular Component	GO:0000314	organellar small ribosomal subunit	1.23E−03	4
GO: Cellular Component	GO:0031597	cytosolic proteasome complex	1.73E−03	3
GO: Cellular Component	GO:0008540	proteasome regulatory particle, base subcomplex	1.73E−03	3
GO: Cellular Component	GO:0016604	nuclear body	1.99E−03	11
GO: Cellular Component	GO:0005916	fascia adherens	1.99E−03	3
GO: Cellular Component	GO:0036464	cytoplasmic ribonucleoprotein granule	1.99E−03	7

GO: Cellular Component	GO:0015934	large ribosomal subunit	2.40E−03	6
GO: Cellular Component	GO:0005730	nucleolus	2.40E−03	18
GO: Cellular Component	GO:0005925	focal adhesion	3.23E−03	11
GO: Cellular Component	GO:0005924	cell-substrate adherens junction	3.46E−03	11
GO: Cellular Component	GO:0043596	nuclear replication fork	3.54E−03	4
GO: Cellular Component	GO:0030055	cell-substrate junction	3.57E−03	11
GO: Cellular Component	GO:0005853	eukaryotic translation elongation factor 1 complex	4.25E−03	2
GO: Cellular Component	GO:0005686	U2 snRNP	4.63E−03	3
GO: Cellular Component	GO:0042645	mitochondrial nucleoid	4.82E−03	4
GO: Cellular Component	GO:0009295	nucleoid	5.50E−03	4
GO: Cellular Component	GO:0005838	proteasome regulatory particle	5.71E−03	3
GO: Cellular Component	GO:0034399	nuclear periphery	6.37E−03	6
GO: Cellular Component	GO:0071013	catalytic step 2 spliceosome	6.53E−03	5
GO: Cellular Component	GO:0022624	proteasome accessory complex	7.83E−03	3
GO: Cellular Component	GO:0017101	aminoacyl-tRNA synthetase multienzyme complex	8.24E−03	2
GO: Cellular Component	GO:0014704	intercalated disc	9.53E−03	4
GO: Cellular Component	GO:0016363	nuclear matrix	1.17E−02	5
GO: Cellular Component	GO:0072669	tRNA-splicing ligase complex	1.41E−02	2
GO: Cellular Component	GO:0030894	replisome	1.47E−02	3
GO: Cellular Component	GO:0005657	replication fork	1.48E−02	4
GO: Cellular Component	GO:0044427	chromosomal part	1.64E−02	15
GO: Cellular Component	GO:0005915	zonula adherens	1.64E−02	2
GO: Cellular Component	GO:0044291	cell-cell contact zone	1.71E−02	4
GO: Cellular Component	GO:0010494	cytoplasmic stress granule	1.71E−02	3
GO: Cellular Component	GO:0000502	proteasome complex	2.30E−02	4
GO: Cellular Component	GO:0000313	organellar ribosome	2.31E−02	4
GO: Cellular Component	GO:0005761	mitochondrial ribosome	2.31E−02	4
GO: Cellular Component	GO:0000784	nuclear chromosome, telomeric region	2.57E−02	5
GO: Cellular Component	GO:0005759	mitochondrial matrix	3.86E−02	9
GO: Cellular Component	GO:0005694	chromosome	3.94E−02	15
GO: Cellular Component	GO:0005662	DNA replication factor A complex	4.38E−02	2
GO: Cellular Component	GO:0043626	PCNA complex	4.74E−02	1
GO: Cellular Component	GO:1990298	bub1-bub3 complex	4.74E−02	1
GO: Cellular Component	GO:0033597	mitotic checkpoint complex	4.74E−02	1
GO: Cellular Component	GO:0071665	gamma-catenin-TCF7L2 complex	4.74E−02	1
GO: Cellular Component	GO:0044796	DNA polymerase processivity factor complex	4.74E−02	1
GO: Cellular Component	GO:0097525	spliceosomal snRNP complex	4.89E−02	3
GO: Cellular Component	GO:0000781	chromosome, telomeric region	4.89E−02	5
**KAP1-interacting proteins *increased* in the presence of SMURF2 wild-type**				
**Molecular Functions – None identified**				
**Biological Processes – None identified**				
**Cellular Component**				
GO: Cellular Component	GO:0000781	chromosome, telomeric region	1.04E−02	4
**KAP1-interacting proteins *decreased* in the presence of SMURF2 wild-type**				
**Molecular Functions**				
GO: Molecular Function	GO:0003723	RNA binding	4.61E−08	22
**Biological Processes**				
GO: Biological Process	GO:0046726	positive regulation by virus of viral protein levels in host cell	4.20E−02	2
GO: Biological Process	GO:0043604	amide biosynthetic process	4.20E−02	10
GO: Biological Process	GO:0006412	translation	4.20E−02	9

GO: Biological Process	GO:0008380	RNA splicing	4.20E−02	7
GO: Biological Process	GO:0043043	peptide biosynthetic process	4.20E−02	9
GO: Biological Process	GO:1901566	organonitrogen compound biosynthetic process	4.20E−02	13
		**Cellular Component**		
GO: Cellular Component	GO:0034399	nuclear periphery	9.47E−03	5
GO: Cellular Component	GO:1990904	ribonucleoprotein complex	1.81E−02	9
GO: Cellular Component	GO:0016363	nuclear matrix	1.81E−02	4
GO: Cellular Component	GO:0005925	focal adhesion	2.97E−02	6
GO: Cellular Component	GO:0005924	cell-substrate adherens junction	2.97E−02	6
GO: Cellular Component	GO:0030055	cell-substrate junction	2.97E−02	6
GO: Cellular Component	GO:0005739	mitochondrion	2.97E−02	13
GO: Cellular Component	GO:0019866	organelle inner membrane	2.97E−02	7
GO: Cellular Component	GO:0015935	small ribosomal subunit	3.08E−02	3
GO: Cellular Component	GO:0005686	U2 snRNP	3.30E−02	2
GO: Cellular Component	GO:0044427	chromosomal part	3.48E−02	8
GO: Cellular Component	GO:0005912	adherens junction	3.48E−02	6
GO: Cellular Component	GO:0098687	chromosomal region	3.48E−02	5
GO: Cellular Component	GO:0070161	anchoring junction	3.48E−02	6
GO: Cellular Component	GO:0005743	mitochondrial inner membrane	3.48E−02	6
GO: Cellular Component	GO:0000314	organellar small ribosomal subunit	3.48E−02	2
GO: Cellular Component	GO:0005763	mitochondrial small ribosomal subunit	3.48E−02	2
GO: Cellular Component	GO:1990298	bub1-bub3 complex	3.48E−02	1
GO: Cellular Component	GO:0043626	PCNA complex	3.48E−02	1
GO: Cellular Component	GO:0033597	mitotic checkpoint complex	3.48E−02	1
GO: Cellular Component	GO:0044796	DNA polymerase processivity factor complex	3.48E−02	1
GO: Cellular Component	GO:0005684	U2-type spliceosomal complex	3.82E−02	2
GO: Cellular Component	GO:0030894	replisome	3.82E−02	2
GO: Cellular Component	GO:0000307	cyclin-dependent protein kinase holoenzyme complex	3.89E−02	2
GO: Cellular Component	GO:0008180	COP9 signalosome	4.05E−02	2
GO: Cellular Component	GO:0010494	cytoplasmic stress granule	4.05E−02	2
GO: Cellular Component	GO:0005694	chromosome	4.09E−02	8
GO: Cellular Component	GO:0031967	organelle envelope	4.25E−02	9
GO: Cellular Component	GO:0031975	envelope	4.25E−02	9
GO: Cellular Component	GO:0014801	longitudinal sarcoplasmic reticulum	4.32E−02	1
GO: Cellular Component	GO:1990630	IRE1-RACK1-PP2A complex	4.32E−02	1
GO: Cellular Component	GO:0090534	calcium ion-transporting ATPase complex	4.32E−02	1
GO: Cellular Component	GO:0070557	PCNA-p21 complex	4.32E−02	1
GO: Cellular Component	GO:0044429	mitochondrial part	4.32E−02	8
GO: Cellular Component	GO:0043596	nuclear replication fork	4.34E−02	2
GO: Cellular Component	GO:0000784	nuclear chromosome, telomeric region	4.39E−02	3
GO: Cellular Component	GO:0005759	mitochondrial matrix	4.51E−02	5
GO: Cellular Component	GO:0030496	midbody	4.51E−02	3
GO: Cellular Component	GO:0042645	mitochondrial nucleoid	4.88E−02	2
		**KAP1-interacting proteins *increased* in the presence of SMURF2 mutant**		
		**Molecular Functions**		
GO: Molecular Function	GO:0003723	RNA binding	1.35E−11	22
GO: Molecular Function	GO:0003735	structural constituent of ribosome	6.16E−08	9
GO: Molecular Function	GO:0019843	rRNA binding	2.23E−05	5
GO: Molecular Function	GO:0003729	mRNA binding	1.12E−04	6
GO: Molecular Function	GO:0031072	heat shock protein binding	1.32E−04	5
GO: Molecular Function	GO:0005198	structural molecule activity	8.79E−04	9
GO: Molecular Function	GO:0051082	unfolded protein binding	2.21E−03	4
GO: Molecular Function	GO:0097157	pre-mRNA intronic binding	3.27E−03	2

GO: Molecular Function	GO:0070181	small ribosomal subunit rRNA binding	3.27E−03	2
GO: Molecular Function	GO:0030955	potassium ion binding	1.09E−02	2
GO: Molecular Function	GO:0031420	alkali metal ion binding	2.37E−02	2
GO: Molecular Function	GO:0036002	pre-mRNA binding	2.57E−02	2
GO: Molecular Function	GO:0060590	ATPase regulator activity	3.26E−02	2
GO: Molecular Function	GO:0015367	oxoglutarate:malate antiporter activity	3.26E−02	1
GO: Molecular Function	GO:0097158	pre-mRNA intronic pyrimidine-rich binding	3.26E−02	1
GO: Molecular Function	GO:0032358	oxidized pyrimidine DNA binding	3.26E−02	1
GO: Molecular Function	GO:0043531	ADP binding	4.73E−02	2
GO: Molecular Function	GO:0030554	adenyl nucleotide binding	4.73E−02	9
GO: Molecular Function	GO:0047066	phospholipid-hydroperoxide glutathione peroxidase activity	4.73E−02	1
GO: Molecular Function	GO:0004743	pyruvate kinase activity	4.73E−02	1
GO: Molecular Function	GO:0005055	laminin receptor activity	4.73E−02	1
GO: Molecular Function	GO:0015140	malate transmembrane transporter activity	4.73E−02	1
		**Biological Processes**		
GO: Biological Process	GO:0006614	SRP-dependent cotranslational protein targeting to membrane	5.77E−07	7
GO: Biological Process	GO:0006613	cotranslational protein targeting to membrane	5.77E−07	7
GO: Biological Process	GO:0045047	protein targeting to ER	5.77E−07	7
GO: Biological Process	GO:0072599	establishment of protein localization to endoplasmic reticulum	5.77E−07	7
GO: Biological Process	GO:0000184	nuclear-transcribed mRNA catabolic process, nonsense-mediated decay	8.16E−07	7
GO: Biological Process	GO:0006413	translational initiation	8.16E−07	8
GO: Biological Process	GO:0016071	mRNA metabolic process	8.16E−07	12
GO: Biological Process	GO:0006412	translation	8.16E−07	12
GO: Biological Process	GO:0070972	protein localization to endoplasmic reticulum	8.16E−07	7
GO: Biological Process	GO:0006518	peptide metabolic process	9.69E−07	13
GO: Biological Process	GO:0043043	peptide biosynthetic process	1.05E−06	12
GO: Biological Process	GO:0006396	RNA processing	1.97E−06	13
GO: Biological Process	GO:0043604	amide biosynthetic process	3.13E−06	12
GO: Biological Process	GO:0006364	rRNA processing	4.31E−06	8
GO: Biological Process	GO:0016072	rRNA metabolic process	4.90E−06	8
GO: Biological Process	GO:0019083	viral transcription	4.90E−06	7
GO: Biological Process	GO:0043603	cellular amide metabolic process	5.31E−06	13
GO: Biological Process	GO:0019080	viral gene expression	6.60E−06	7
GO: Biological Process	GO:0006612	protein targeting to membrane	8.03E−06	7
GO: Biological Process	GO:0034655	nucleobase-containing compound catabolic process	8.52E−06	9
GO: Biological Process	GO:0000956	nuclear-transcribed mRNA catabolic process	9.57E−06	7
GO: Biological Process	GO:0044033	multi-organism metabolic process	1.08E−05	7
GO: Biological Process	GO:0042254	ribosome biogenesis	1.35E−05	8
GO: Biological Process	GO:0006402	mRNA catabolic process	1.40E−05	7
GO: Biological Process	GO:0044270	cellular nitrogen compound catabolic process	1.58E−05	9
GO: Biological Process	GO:0046700	heterocycle catabolic process	1.58E−05	9
GO: Biological Process	GO:0019439	aromatic compound catabolic process	1.82E−05	9
GO: Biological Process	GO:1901566	organonitrogen compound biosynthetic process	2.49E−05	14
GO: Biological Process	GO:0006401	RNA catabolic process	2.70E−05	7
GO: Biological Process	GO:1901361	organic cyclic compound catabolic process	3.02E−05	9
GO: Biological Process	GO:0072594	establishment of protein localization to organelle	3.23E−05	10
GO: Biological Process	GO:0034470	ncRNA processing	4.90E−05	8
GO: Biological Process	GO:0006605	protein targeting	5.58E−05	10
GO: Biological Process	GO:0033365	protein localization to organelle	6.05E−05	11
GO: Biological Process	GO:0019058	viral life cycle	1.04E−04	8
GO: Biological Process	GO:0034605	cellular response to heat	1.12E−04	5
GO: Biological Process	GO:0022613	ribonucleoprotein complex biogenesis	1.38E−04	8
GO: Biological Process	GO:0006457	protein folding	2.59E−04	6

GO: Biological Process	GO:0000028	ribosomal small subunit assembly	2.67E−04	3
GO: Biological Process	GO:0006886	intracellular protein transport	3.02E−04	11
GO: Biological Process	GO:0090150	establishment of protein localization to membrane	3.11E−04	7
GO: Biological Process	GO:0042026	protein refolding	3.83E−04	3
GO: Biological Process	GO:0034660	ncRNA metabolic process	4.57E−04	8
GO: Biological Process	GO:0016032	viral process	5.22E−04	9
GO: Biological Process	GO:0044265	cellular macromolecule catabolic process	5.77E−04	10
GO: Biological Process	GO:0044764	multi-organism cellular process	5.77E−04	9
GO: Biological Process	GO:0009408	response to heat	6.45E−04	5
GO: Biological Process	GO:0009057	macromolecule catabolic process	6.51E−04	11
GO: Biological Process	GO:0044419	interspecies interaction between organisms	7.52E−04	9
GO: Biological Process	GO:0044403	symbiont process	7.52E−04	9
GO: Biological Process	GO:0010608	posttranscriptional regulation of gene expression	1.10E−03	7
GO: Biological Process	GO:0006417	regulation of translation	1.65E−03	6
GO: Biological Process	GO:0072657	protein localization to membrane	1.76E−03	7
GO: Biological Process	GO:0034613	cellular protein localization	2.04E−03	12
GO: Biological Process	GO:0070727	cellular macromolecule localization	2.12E−03	12
GO: Biological Process	GO:0009266	response to temperature stimulus	2.47E−03	5
GO: Biological Process	GO:0034248	regulation of cellular amide metabolic process	2.47E−03	6
GO: Biological Process	GO:0006414	translational elongation	3.25E−03	4
GO: Biological Process	GO:0008380	RNA splicing	3.25E−03	6
GO: Biological Process	GO:0042255	ribosome assembly	4.22E−03	3
GO: Biological Process	GO:0061024	membrane organization	6.02E−03	9
GO: Biological Process	GO:0042274	ribosomal small subunit biogenesis	6.02E−03	3
GO: Biological Process	GO:1901575	organic substance catabolic process	6.37E−03	12
GO: Biological Process	GO:0000377	RNA splicing, via transesterification reactions with bulged adenosine as nucleophile	7.04E−03	5
GO: Biological Process	GO:0000398	mRNA splicing, via spliceosome	7.04E−03	5
GO: Biological Process	GO:0015031	protein transport	7.25E−03	12
GO: Biological Process	GO:0000375	RNA splicing, via transesterification reactions	7.25E−03	5
GO: Biological Process	GO:1900034	regulation of cellular response to heat	8.70E−03	3
GO: Biological Process	GO:0006450	regulation of translational fidelity	9.09E−03	2
GO: Biological Process	GO:0097194	execution phase of apoptosis	1.06E−02	3
GO: Biological Process	GO:0009628	response to abiotic stimulus	1.16E−02	9
GO: Biological Process	GO:0006309	apoptotic DNA fragmentation	1.47E−02	2
GO: Biological Process	GO:0044248	cellular catabolic process	1.49E−02	11
GO: Biological Process	GO:0000737	DNA catabolic process, endonucleolytic	1.98E−02	2
GO: Biological Process	GO:0006448	regulation of translational elongation	1.98E−02	2
GO: Biological Process	GO:0045727	positive regulation of translation	2.05E−02	3
GO: Biological Process	GO:1900117	regulation of execution phase of apoptosis	2.08E−02	2
GO: Biological Process	GO:0043484	regulation of RNA splicing	2.16E−02	3
GO: Biological Process	GO:0033119	negative regulation of RNA splicing	2.48E−02	2
GO: Biological Process	GO:0048147	negative regulation of fibroblast proliferation	2.48E−02	2
GO: Biological Process	GO:0030262	apoptotic nuclear changes	2.48E−02	2
GO: Biological Process	GO:1905051	regulation of base-excision repair	2.48E−02	1
GO: Biological Process	GO:1904045	cellular response to aldosterone	2.48E−02	1
GO: Biological Process	GO:1902544	regulation of DNA N-glycosylase activity	2.48E−02	1
GO: Biological Process	GO:1905053	positive regulation of base-excision repair	2.48E−02	1
GO: Biological Process	GO:0090297	positive regulation of mitochondrial DNA replication	2.48E−02	1
GO: Biological Process	GO:1900208	regulation of cardiolipin metabolic process	2.48E−02	1
GO: Biological Process	GO:1902546	positive regulation of DNA N-glycosylase activity	2.48E−02	1
GO: Biological Process	GO:1900210	positive regulation of cardiolipin metabolic process	2.48E−02	1
GO: Biological Process	GO:0061481	response to TNF agonist	2.48E−02	1

GO: Biological Process	GO:2001272	positive regulation of cysteine-type endopeptidase activity involved in execution phase of apoptosis	2.48E−02	1
GO: Biological Process	GO:1904044	response to aldosterone	2.48E−02	1
GO: Biological Process	GO:1990046	stress-induced mitochondrial fusion	2.48E−02	1
GO: Biological Process	GO:0034250	positive regulation of cellular amide metabolic process	2.65E−02	3
GO: Biological Process	GO:0006308	DNA catabolic process	2.66E−02	2
GO: Biological Process	GO:0022411	cellular component disassembly	2.73E−02	6
GO: Biological Process	GO:0042769	DNA damage response, detection of DNA damage	2.89E−02	2
GO: Biological Process	GO:0000381	regulation of alternative mRNA splicing, via spliceosome	2.89E−02	2
GO: Biological Process	GO:0006397	mRNA processing	3.43E−02	5
GO: Biological Process	GO:0006913	nucleocytoplasmic transport	3.88E−02	5
GO: Biological Process	GO:0051169	nuclear transport	4.11E−02	5
GO: Biological Process	GO:1901860	positive regulation of mitochondrial DNA metabolic process	4.39E−02	1
GO: Biological Process	GO:0006880	intracellular sequestering of iron ion	4.39E−02	1
GO: Biological Process	GO:0097577	sequestering of iron ion	4.39E−02	1
GO: Biological Process	GO:0000461	endonucleolytic cleavage to generate mature 3'-end of SSU-rRNA from (SSU-rRNA, 5.8S rRNA, LSU-rRNA)	4.39E−02	1
GO: Biological Process	GO:0000380	alternative mRNA splicing, via spliceosome	4.52E−02	2
GO: Biological Process	GO:0006754	ATP biosynthetic process	4.67E−02	2
		**Cellular Component**		
GO: Cellular Component	GO:0015935	small ribosomal subunit	2.59E−10	8
GO: Cellular Component	GO:0022627	cytosolic small ribosomal subunit	2.59E−10	7
GO: Cellular Component	GO:1990904	ribonucleoprotein complex	5.86E−09	14
GO: Cellular Component	GO:0044391	ribosomal subunit	8.72E−08	8
GO: Cellular Component	GO:0022626	cytosolic ribosome	1.36E−07	7
GO: Cellular Component	GO:0005840	ribosome	5.87E−07	8
GO: Cellular Component	GO:0044445	cytosolic part	1.05E−05	7
GO: Cellular Component	GO:0019866	organelle inner membrane	3.21E−04	8
GO: Cellular Component	GO:0005739	mitochondrion	5.47E−04	13
GO: Cellular Component	GO:0008180	COP9 signalosome	7.62E−04	3
GO: Cellular Component	GO:0005730	nucleolus	9.30E−04	9
GO: Cellular Component	GO:0005743	mitochondrial inner membrane	9.30E−04	7
GO: Cellular Component	GO:0044429	mitochondrial part	1.73E−03	9
GO: Cellular Component	GO:0031966	mitochondrial membrane	5.12E−03	7
GO: Cellular Component	GO:0031967	organelle envelope	5.12E−03	9
GO: Cellular Component	GO:0031975	envelope	5.12E−03	9
GO: Cellular Component	GO:0005740	mitochondrial envelope	6.42E−03	7
GO: Cellular Component	GO:0005925	focal adhesion	1.05E−02	5
GO: Cellular Component	GO:0005924	cell-substrate adherens junction	1.05E−02	5
GO: Cellular Component	GO:0005689	U12-type spliceosomal complex	1.05E−02	2
GO: Cellular Component	GO:0030055	cell-substrate junction	1.05E−02	5
GO: Cellular Component	GO:0005912	adherens junction	2.22E−02	5
GO: Cellular Component	GO:0072562	blood microparticle	2.22E−02	3
GO: Cellular Component	GO:0070161	anchoring junction	2.22E−02	5
GO: Cellular Component	GO:0035770	ribonucleoprotein granule	2.22E−02	3
GO: Cellular Component	GO:0044279	other organism membrane	2.22E−02	1
GO: Cellular Component	GO:0044218	other organism cell membrane	2.22E−02	1
GO: Cellular Component	GO:0071159	NF-kappaB complex	2.22E−02	1
GO: Cellular Component	GO:0097165	nuclear stress granule	2.22E−02	1
GO: Cellular Component	GO:0070288	ferritin complex	2.22E−02	1
GO: Cellular Component	GO:0008043	intracellular ferritin complex	2.22E−02	1
GO: Cellular Component	GO:0030532	small nuclear ribonucleoprotein complex	3.63E−02	2
GO: Cellular Component	GO:0043209	myelin sheath	4.12E−02	3
		**KAP1-interacting proteins *decreased* in the presence of SMURF2 mutant**		

		**Molecular Functions**		
GO: Molecular Function	GO:0003723	RNA binding	5.48E−05	15
GO: Molecular Function	GO:0086083	cell adhesive protein binding involved in bundle of His cell-Purkinje myocyte communication	5.91E−03	2
GO: Molecular Function	GO:0005198	structural molecule activity	8.01E−03	8
GO: Molecular Function	GO:0086080	protein binding involved in heterotypic cell-cell adhesion	8.81E−03	2
GO: Molecular Function	GO:0098632	cell-cell adhesion mediator activity	1.03E−02	2
GO: Molecular Function	GO:0098631	cell adhesion mediator activity	1.98E−02	2
GO: Molecular Function	GO:0030507	spectrin binding	4.00E−02	2
GO: Molecular Function	GO:0070626	(S)-2-(5-amino-1-(5-phospho-D-ribosyl)imidazole-4-carboxamido)succinate AMP-lyase (fumarate-forming) activity	4.00E−02	1
GO: Molecular Function	GO:0005199	structural constituent of cell wall	4.00E−02	1
GO: Molecular Function	GO:0004018	N6-(1,2-dicarboxyethyl)AMP AMP-lyase (fumarate-forming) activity	4.00E−02	1
		**Biological Processes**		
GO: Biological Process	GO:0016071	mRNA metabolic process	1.55E−02	8
GO: Biological Process	GO:0086073	bundle of His cell-Purkinje myocyte adhesion involved in cell communication	1.55E−02	2
GO: Biological Process	GO:0022618	ribonucleoprotein complex assembly	1.55E−02	5
GO: Biological Process	GO:0006396	RNA processing	1.55E−02	9
GO: Biological Process	GO:0071826	ribonucleoprotein complex subunit organization	1.55E−02	5
GO: Biological Process	GO:0086042	cardiac muscle cell-cardiac muscle cell adhesion	1.55E−02	2
GO: Biological Process	GO:0002934	desmosome organization	2.42E−02	2
GO: Biological Process	GO:0065003	protein-containing complex assembly	2.42E−02	12
GO: Biological Process	GO:0086069	bundle of His cell to Purkinje myocyte communication	2.42E−02	2
GO: Biological Process	GO:0098911	regulation of ventricular cardiac muscle cell action potential	2.42E−02	2
GO: Biological Process	GO:0022613	ribonucleoprotein complex biogenesis	3.21E−02	6
GO: Biological Process	GO:0006412	translation	3.21E−02	7
GO: Biological Process	GO:0006397	mRNA processing	3.25E−02	6
GO: Biological Process	GO:0090136	epithelial cell-cell adhesion	3.28E−02	2
GO: Biological Process	GO:0043043	peptide biosynthetic process	3.32E−02	7
GO: Biological Process	GO:0006413	translational initiation	4.38E−02	4
GO: Biological Process	GO:0034622	cellular protein-containing complex assembly	4.59E−02	8
GO: Biological Process	GO:0098901	regulation of cardiac muscle cell action potential	4.59E−02	2
		**Cellular Component**		
GO: Cellular Component	GO:0005916	fascia adherens	3.63E−04	3
GO: Cellular Component	GO:0005913	cell-cell adherens junction	8.78E−04	4
GO: Cellular Component	GO:0005915	zonula adherens	8.10E−03	2
GO: Cellular Component	GO:0014704	intercalated disc	9.20E−03	3
GO: Cellular Component	GO:0005912	adherens junction	1.11E−02	6
GO: Cellular Component	GO:0044291	cell-cell contact zone	1.11E−02	3
GO: Cellular Component	GO:0070161	anchoring junction	1.11E−02	6
GO: Cellular Component	GO:1990904	ribonucleoprotein complex	1.31E−02	7
GO: Cellular Component	GO:0030057	desmosome	2.39E−02	2
GO: Cellular Component	GO:0071665	gamma-catenin-TCF7L2 complex	3.52E−02	1

Table S5. Details of the Ingenuity Pathway Analysis (IPA) of KAP1 interactome affected by SMURF2WT and its catalytically-inactive mutant (SMURF2CG). The data are sorted based on −log(p-value) (from high-to-lower). Negative Z-score value predicts pathway inhibition, whereas positive Z-score indicates predicted pathway activation. NaN—Z-score cannot be calculated for Ingenuity canonical pathways.

**Enriched for SMURF2WT vs. Empty**				
**Ingenuity Canonical Pathways**	**−log (*p*-value)**	**z-score**	**Downregulated/Total Number of Proteins in the Corresponding Signaling Pathway**	**Upregulated/Total Number of Proteins in the Corresponding Signaling Pathway**
EIF2 Signaling	81.6	−5.571	71/227 (31%)	17/227 (7%)
Regulation of eIF4 and p70S6K Signaling	26	−1	27/183 (15%)	13/183 (7%)
Coronavirus Pathogenesis Pathway	22.1	2.596	28/203 (14%)	10/203 (5%)
mTOR Signaling	21.9	−1.342	27/218 (12%)	12/218 (6%)
BAG2 Signaling Pathway	18.5	−1.941	17/86 (20%)	7/86 (8%)
Protein Ubiquitination Pathway	16.3	NaN	22/279 (8%)	15/279 (5%)
Huntington's Disease Signaling	10.6	0.447	20/289 (7%)	10/289 (3%)
Coronavirus Replication Pathway	8.01	−2.714	10/46 (22%)	1/46 (2%)
FAT10 Signaling Pathway	7.83	NaN	8/59 (14%)	4/59 (7%)
Inhibition of ARE-Mediated mRNA Degradation Pathway	7.83	−1.414	13/161 (8%)	6/161 (4%)
Unfolded protein response	7.45	−2.828	10/90 (11%)	4/90 (4%)
Sirtuin Signaling Pathway	6.34	−2.357	20/325 (6%)	5/325 (2%)
Role of PKR in Interferon Induction and Antiviral Response	5.89	0.775	13/137 (9%)	2/137 (1%)
Aldosterone Signaling in Epithelial Cells	5.89	NaN	10/174 (6%)	7/174 (4%)
RAN Signaling	5.38	0	3/19 (16%)	3/19 (16%)
Remodeling of Epithelial Adherens Junctions	5.27	NaN	9/68 (13%)	1/68 (1%)
ATM Signaling	4.54	−1.414	6/99 (6%)	5/99 (5%)
Cyclins and Cell Cycle Regulation	4.44	1.414	4/84 (5%)	6/84 (7%)
Cell Cycle: G1/S Checkpoint Regulation	4.42	−2.828	6/68 (9%)	3/68 (4%)
CSDE1 Signaling Pathway	4.23	−1.414	7/56 (13%)	1/56 (2%)
HIF1α Signaling	4.13	2.84	12/214 (6%)	4/214 (2%)
Germ Cell-Sertoli Cell Junction Signaling	4.03	NaN	11/174 (6%)	3/174 (2%)
Mitotic Roles of Polo-Like Kinase	3.72	NaN	2/66 (3%)	6/66 (9%)
Cell Cycle: G2/M DNA Damage Checkpoint Regulation	3.71	−1.342	6/50 (12%)	1/50 (2%)
NRF2-mediated Oxidative Stress Response	3.62	−2.646	8/237 (3%)	8/237 (3%)
Sertoli Cell-Sertoli Cell Junction Signaling	3.6	NaN	12/214 (6%)	3/214 (1%)
Spliceosomal Cycle	3.55	0.378	3/53 (6%)	4/53 (8%)
DNA Double-Strand Break Repair by Non-Homologous End Joining	3.55	NaN	3/14 (21%)	1/14 (7%)
14-3-3-mediated Signaling	3.5	NaN	11/129 (9%)	0/129 (0%)
Telomere Extension by Telomerase	3.42	NaN	2/15 (13%)	2/15 (13%)
Role of CHK Proteins in Cell Cycle Checkpoint Control	3.35	−0.816	2/57 (4%)	5/57 (9%)
eNOS Signaling	2.98	2.333	8/172 (5%)	4/172 (2%)
DNA Methylation and Transcriptional Repression Signaling	2.83	NaN	1/35 (3%)	4/35 (11%)
Cell Cycle Regulation by BTG Family Proteins	2.72	NaN	1/37 (3%)	4/37 (11%)
Necroptosis Signaling Pathway	2.7	−2.111	10/161 (6%)	1/161 (1%)
Phagosome Maturation	2.6	NaN	11/166 (7%)	0/166 (0%)
WNT/β-catenin Signaling	2.44	−1.134	6/174 (3%)	5/174 (3%)
BER (Base Excision Repair) Pathway	2.39	−0.447	2/44 (5%)	3/44 (7%)
Role of p14/p19ARF in Tumor Suppression	2.3	NaN	4/29 (14%)	0/29 (0%)
p53 Signaling	2	−2	4/98 (4%)	3/98 (3%)
Granzyme A Signaling	1.99	NaN	3/19 (16%)	0/19 (0%)
ILK Signaling	1.97	0.632	4/202 (2%)	7/202 (3%)
Cell Cycle Control of Chromosomal Replication	1.94	1.342	1/56 (2%)	4/56 (7%)
Estrogen Receptor Signaling	1.89	0.277	9/413 (2%)	9/413 (2%)
Cysteine Biosynthesis III (mammalia)	1.88	1	1/38 (3%)	3/38 (8%)

Pyrimidine Ribonucleotides De Novo Biosynthesis	1.85	−1.342	4/59 (7%)	1/59 (2%)
Gap Junction Signaling	1.84	NaN	9/211 (4%)	2/211 (1%)
Telomerase Signaling	1.81	0.816	2/107 (2%)	5/107 (5%)
Mitochondrial Dysfunction	1.77	NaN	5/188 (3%)	5/188 (3%)
HIPPO signaling	1.74	0.447	4/86 (5%)	2/86 (2%)
p70S6K Signaling	1.71	−1.633	6/138 (4%)	2/138 (1%)
Tetrahydrofolate Salvage from 5,10-methenyltetrahydrofolate	1.63	NaN	1/10 (10%)	1/10 (10%)
Estrogen-mediated S-phase Entry	1.61	NaN	0/26 (0%)	3/26 (12%)
Hereditary Breast Cancer Signaling	1.61	NaN	3/144 (2%)	5/144 (3%)
Pyrimidine Ribonucleotides Interconversion	1.6	−2	4/46 (9%)	0/46 (0%)
Natural Killer Cell Signaling	1.56	−2.53	10/204 (5%)	0/204 (0%)
Oxidative Phosphorylation	1.55	0.378	3/121 (2%)	4/121 (3%)
Glucocorticoid Receptor Signaling	1.49	NaN	16/591 (3%)	6/591 (1%)
Methionine Degradation I (to Homocysteine)	1.41	NaN	1/31 (3%)	2/31 (6%)
Transcriptional Regulatory Network in Embryonic Stem Cells	1.38	NaN	3/54 (6%)	1/54 (2%)
Xenobiotic Metabolism CAR Signaling Pathway	1.37	1.667	2/190 (1%)	7/190 (4%)
Toll-like Receptor Signaling	1.37	NaN	4/79 (5%)	1/79 (1%)
Ferroptosis Signaling Pathway	1.35	−1.414	4/162 (2%)	4/162 (2%)
Aryl Hydrocarbon Receptor Signaling	1.31	0	2/165 (1%)	6/165 (4%)
Sumoylation Pathway	1.3	−1.342	2/109 (2%)	4/109 (4%)

**Enriched for SMURF2WT vs. SMURF2CG**				
**Ingenuity Canonical Pathways**	**−log (*p*-value)**	**z-score**	**Downregulated/Total Number of Proteins in the Corresponding Signaling Pathway**	**Upregulated/Total Number of Proteins in the Corresponding Signaling Pathway**
EIF2 Signaling	84.4	−3	66/227 (29%)	22/227 (10%)
Regulation of eIF4 and p70S6K Signaling	27.1	−1	30/183 (16%)	10/183 (5%)
mTOR Signaling	23	−1.342	30/218 (14%)	9/218 (4%)
Coronavirus Pathogenesis Pathway	22.2	3.781	31/203 (15%)	6/203 (3%)
BAG2 Signaling Pathway	19.2	−1.941	18/86 (21%)	6/86 (7%)
Protein Ubiquitination Pathway	15.6	NaN	25/279 (9%)	10/279 (4%)
Huntington's Disease Signaling	11.3	1.342	22/289 (8%)	8/289 (3%)
Coronavirus Replication Pathway	8.33	−2.714	10/46 (22%)	1/46 (2%)
Inhibition of ARE-Mediated mRNA Degradation Pathway	8.32	−2.121	11/161 (7%)	8/161 (5%)
FAT10 Signaling Pathway	8.16	NaN	7/59 (12%)	5/59 (8%)
Unfolded protein response	7.83	−2.828	13/90 (14%)	1/90 (1%)
Sirtuin Signaling Pathway	6.91	−2.357	21/325 (6%)	4/325 (1%)
Aldosterone Signaling in Epithelial Cells	6.31	NaN	14/174 (8%)	3/174 (2%)
Role of PKR in Interferon Induction and Antiviral Response	6.27	1.807	13/137 (9%)	2/137 (1%)
RAN Signaling	5.56	−1.633	5/19 (26%)	1/19 (5%)
Remodeling of Epithelial Adherens Junctions	5.54	NaN	9/68 (13%)	1/68 (1%)
HIF1*α* Signaling	4.49	2.84	14/214 (7%)	2/214 (1%)
CSDE1 Signaling Pathway	4.45	−1.414	7/56 (13%)	1/56 (2%)
ATM Signaling	4.08	−0.378	7/99 (7%)	3/99 (3%)
Cyclins and Cell Cycle Regulation	3.92	0.378	6/84 (7%)	3/84 (4%)
Cell Cycle: G2/M DNA Damage Checkpoint Regulation	3.9	−0.447	7/50 (14%)	0/50 (0%)
Cell Cycle: G1/S Checkpoint Regulation	3.83	−1.134	6/68 (9%)	2/68 (3%)
14-3-3-mediated Signaling	3.76	NaN	11/129 (9%)	0/129 (0%)
Germ Cell-Sertoli Cell Junction Signaling	3.75	NaN	11/174 (6%)	2/174 (1%)
DNA Double-Strand Break Repair by Non-Homologous End Joining	3.66	NaN	3/14 (21%)	1/14 (7%)
Telomere Extension by Telomerase	3.54	NaN	4/15 (27%)	0/15 (0%)
NRF2-mediated Oxidative Stress Response	3.45	−2.449	11/237 (5%)	4/237 (2%)

Sertoli Cell-Sertoli Cell Junction Signaling	3.4	NaN	11/214 (5%)	3/214 (1%)
eNOS Signaling	3.24	3	10/172 (6%)	2/172 (1%)
Mitotic Roles of Polo-Like Kinase	3.14	NaN	3/66 (5%)	4/66 (6%)
DNA Methylation and Transcriptional Repression Signaling	2.97	NaN	3/35 (9%)	2/35 (6%)
Necroptosis Signaling Pathway	2.94	−0.905	8/161 (5%)	3/161 (2%)
Phagosome Maturation	2.84	NaN	11/166 (7%)	0/166 (0%)
Role of CHK Proteins in Cell Cycle Checkpoint Control	2.75	0	3/57 (5%)	3/57 (5%)
WNT/β-catenin Signaling	2.67	0.378	8/174 (5%)	3/174 (2%)
Role of p14/p19ARF in Tumor Suppression	2.41	NaN	4/29 (14%)	0/29 (0%)
Spliceosomal Cycle	2.16	−1.342	4/53 (8%)	1/53 (2%)
Granzyme A Signaling	2.07	NaN	3/19 (16%)	0/19 (0%)
Gap Junction Signaling	2.05	NaN	9/211 (4%)	2/211 (1%)
Cell Cycle Regulation by BTG Family Proteins	2.02	NaN	1/37 (3%)	3/37 (8%)
Telomerase Signaling	1.96	0	4/107 (4%)	3/107 (3%)
p70S6K Signaling	1.87	−0.816	5/138 (4%)	3/138 (2%)
HIPPO signaling	1.87	0.447	4/86 (5%)	2/86 (2%)
Estrogen Receptor Signaling	1.87	0	11/413 (3%)	6/413 (1%)
Glucocorticoid Receptor Signaling	1.79	NaN	17/591 (3%)	5/591 (1%)
ILK Signaling	1.77	−0.333	3/202 (1%)	7/202 (3%)
BER (Base Excision Repair) Pathway	1.76	0	3/44 (7%)	1/44 (2%)
Natural Killer Cell Signaling	1.74	−2.53	10/204 (5%)	0/204 (0%)
Pyrimidine Ribonucleotides Interconversion	1.7	−1	3/46 (7%)	1/46 (2%)
Tetrahydrofolate Salvage from 5,10-methenyltetrahydrofolate	1.69	NaN	2/10 (20%)	0/10 (0%)
Toll-like Receptor Signaling	1.47	NaN	4/79 (5%)	1/79 (1%)
Transcriptional Regulatory Network in Embryonic Stem Cells	1.47	NaN	3/54 (6%)	1/54 (2%)
NAD Signaling Pathway	1.41	−0.707	6/169 (4%)	2/169 (1%)
PI3K/AKT Signaling	1.36	−1	5/204 (2%)	4/204 (2%)
Pyrimidine Ribonucleotides De Novo Biosynthesis	1.35	−1	3/59 (5%)	1/59 (2%)
Hereditary Breast Cancer Signaling	1.34	NaN	5/144 (3%)	2/144 (1%)
Granzyme B Signaling	1.3	NaN	1/16 (6%)	1/16 (6%)

**Enriched for SMURF2CG vs. Empty**				
**Ingenuity Canonical Pathways**	**−log (*p*-value)**	**z-score**	**Downregulated/Total Number of Proteins in the Corresponding Signaling Pathway**	**Upregulated/Total Number of Proteins in the Corresponding Signaling Pathway**
EIF2 Signaling	79.3	−2.598	41/227 (18%)	44/227 (19%)
Regulation of eIF4 and p70S6K Signaling	24.7	−1	8/183 (4%)	30/183 (16%)
Coronavirus Pathogenesis Pathway	20.9	−2.333	10/203 (5%)	26/203 (13%)
mTOR Signaling	20.8	−0.447	7/218 (3%)	30/218 (14%)
BAG2 Signaling Pathway	17.8	3.051	6/86 (7%)	17/86 (20%)
Protein Ubiquitination Pathway	14.6	NaN	8/279 (3%)	26/279 (9%)
Huntington's Disease Signaling	10.4	−0.447	7/289 (2%)	22/289 (8%)
Coronavirus Replication Pathway	8.26	2.111	2/46 (4%)	9/46 (20%)
Inhibition of ARE-Mediated mRNA Degradation Pathway	7.44	−1.414	9/161 (6%)	9/161 (6%)
FAT10 Signaling Pathway	7.05	NaN	6/59 (10%)	5/59 (8%)
Unfolded protein response	6.85	2.828	1/90 (1%)	12/90 (13%)
Sirtuin Signaling Pathway	6.78	2.828	4/325 (1%)	21/325 (6%)
Role of PKR in Interferon Induction and Antiviral Response	6.19	−2.324	3/137 (2%)	12/137 (9%)
RAN Signaling	5.52	1.633	1/19 (5%)	5/19 (26%)
Remodeling of Epithelial Adherens Junctions	5.48	NaN	1/68 (1%)	9/68 (13%)
Aldosterone Signaling in Epithelial Cells	4.89	NaN	1/174 (1%)	14/174 (8%)
Spliceosomal Cycle	4.58	1.414	2/53 (4%)	6/53 (11%)
HIF1α Signaling	4.41	−1.807	1/214 (0%)	15/214 (7%)

CSDE1 Signaling Pathway	4.4	−0.707	6/56 (11%)	2/56 (4%)
ATM Signaling	4.02	−1.134	2/99 (2%)	8/99 (8%)
Mitotic Roles of Polo-Like Kinase	3.88	NaN	2/66 (3%)	6/66 (9%)
Cyclins and Cell Cycle Regulation	3.87	−1.89	1/84 (1%)	8/84 (10%)
Cell Cycle: G2/M DNA Damage Checkpoint Regulation	3.86	−0.447	2/50 (4%)	5/50 (10%)
14-3-3-mediated Signaling	3.7	NaN	3/129 (2%)	8/129 (6%)
Germ Cell-Sertoli Cell Junction Signaling	3.69	NaN	2/174 (1%)	11/174 (6%)
DNA Double-Strand Break Repair by Non-Homologous End Joining	3.64	NaN	1/14 (7%)	3/14 (21%)
Telomere Extension by Telomerase	3.51	NaN	0/15 (0%)	4/15 (27%)
NRF2-mediated Oxidative Stress Response	3.38	1.134	4/237 (2%)	11/237 (5%)
Sertoli Cell-Sertoli Cell Junction Signaling	3.34	NaN	4/214 (2%)	10/214 (5%)
Cell Cycle: G1/S Checkpoint Regulation	3.03	0.816	2/68 (3%)	5/68 (7%)
Necroptosis Signaling Pathway	2.89	−0.302	5/161 (3%)	6/161 (4%)
Phagosome Maturation	2.79	NaN	3/166 (2%)	8/166 (5%)
Role of CHK Proteins in Cell Cycle Checkpoint Control	2.71	1.342	1/57 (2%)	5/57 (9%)
eNOS Signaling	2.66	−3	0/172 (0%)	11/172 (6%)
WNT/β-catenin Signaling	2.62	−1.89	2/174 (1%)	9/174 (5%)
BER (Base Excision Repair) Pathway	2.49	−0.447	2/44 (5%)	3/44 (7%)
Role of p14/p19ARF in Tumor Suppression	2.38	NaN	1/29 (3%)	3/29 (10%)
Mitochondrial Dysfunction	2.37	NaN	2/188 (1%)	9/188 (5%)
p53 Signaling	2.13	1	3/98 (3%)	4/98 (4%)
Granzyme A Signaling	2.05	NaN	1/19 (5%)	2/19 (11%)
Cell Cycle Regulation by BTG Family Proteins	2	NaN	1/37 (3%)	3/37 (8%)
Telomerase Signaling	1.93	−0.816	0/107 (0%)	7/107 (7%)
HIPPO signaling	1.84	−0.447	2/86 (2%)	4/86 (5%)
p70S6K Signaling	1.84	−0.816	3/138 (2%)	5/138 (4%)
Estrogen Receptor Signaling	1.81	1.732	2/413 (0%)	15/413 (4%)
Hereditary Breast Cancer Signaling	1.74	NaN	3/144 (2%)	5/144 (3%)
ILK Signaling	1.73	0.333	3/202 (1%)	7/202 (3%)
Glucocorticoid Receptor Signaling	1.72	NaN	2/591 (0%)	20/591 (3%)
Natural Killer Cell Signaling	1.7	2.53	0/204 (0%)	10/204 (5%)
Tetrahydrofolate Salvage from 5,10-methenyltetrahydrofolate	1.68	NaN	1/10 (10%)	1/10 (10%)
Oxidative Phosphorylation	1.66	1.89	1/121 (1%)	6/121 (5%)
Gap Junction Signaling	1.61	NaN	1/211 (0%)	9/211 (4%)
Xenobiotic Metabolism CAR Signaling Pathway	1.49	2.333	1/190 (1%)	8/190 (4%)
Ferroptosis Signaling Pathway	1.47	−1.414	1/162 (1%)	7/162 (4%)
Toll-like Receptor Signaling	1.45	NaN	3/79 (4%)	2/79 (3%)
AMPK Signaling	1.41	−1.265	4/260 (2%)	7/260 (3%)
Cell Cycle Control of Chromosomal Replication	1.4	0	2/56 (4%)	2/56 (4%)
Sumoylation Pathway	1.4	0.447	2/109 (2%)	4/109 (4%)
NAD Signaling Pathway	1.38	0.707	2/169 (1%)	6/169 (4%)
DNA Methylation and Transcriptional Repression Signaling	1.34	NaN	0/35 (0%)	3/35 (9%)
Pyrimidine Ribonucleotides De Novo Biosynthesis	1.33	2	0/59 (0%)	4/59 (7%)
PI3K/AKT Signaling	1.33	−1	3/204 (1%)	6/204 (3%)
GADD45 Signaling	1.31	0	2/60 (3%)	2/60 (3%)
